# IL-2 and TCR stimulation induce expression and secretion of IL-32β by human T cells

**DOI:** 10.3389/fimmu.2024.1437224

**Published:** 2024-08-15

**Authors:** Franziska Christine Sanna, Iva Benešová, Philip Pervan, Adriana Krenz, Alexander Wurzel, Robert Lohmayer, Jasmin Mühlbauer, Amélie Wöllner, Nina Köhl, Ayse Nur Menevse, Slava Stamova, Valentina Volpin, Philipp Beckhove, Maria Xydia

**Affiliations:** ^1^ Interventional Immunology, Leibniz Institute for Immunotherapy, Regensburg, Germany; ^2^ Algorithmic Bioinformatics, Leibniz Institute for Immunotherapy, Regensburg, Germany; ^3^ Department of Internal Medicine III, Hematology and Medical Oncology, University Medical Center, Regensburg, Germany; ^4^ Bavarian Cancer Research Center (BZKF), Regensburg, Germany; ^5^ Department of Biosystems Science and Engineering, ETH Zurich, Basel, Switzerland

**Keywords:** IL-32 isoform, IL-32β, IL-32 secretion mechanism, T cells, exosomes, unconventional secretion pathway, IL-2, cancer

## Abstract

IL-32 expression is important for pathogen clearance but detrimental in chronic inflammation, autoimmunity, and cancer. T cells are major IL-32 producers in these diseases and key mediators of pathogen and tumor elimination but also autoimmune destruction. However, their contribution to IL-32 biology during immune responses is hardly understood due to several isoforms with divergent inflammatory properties. Here, we identified IL-32β as the predominant isoform in various T cell subsets of healthy individuals and breast cancer patients with the highest levels detected in intratumoral regulatory T cells. We show that IL-32β is induced by IL-2 but IL-32β release requires T Cell Receptor rather than IL2R stimulation. Using inhibitors of protein secretion pathways and serial (ultra)centrifugation of T cell supernatants, we demonstrate that T cells actively secrete IL-32β unconventionally, as a free protein and, to a minor degree, through exosomes. Thus, our data identify activated T cells as major IL-32β secretors in health and cancer.

## Introduction

Interleukin-32 (IL-32, NK4) is an unconventional inflammatory cytokine with a yet poorly understood multifaceted role in human health and disease (1). It is produced by a broad variety of cell types, including epithelial and endothelial cells, fibroblasts, cancer cells and immune cells (2). IL-32 expression is induced during infection, conditions of cellular stress, like hypoxia, and by pro-inflammatory cytokines, like IFN-γ and TNF-α (2), thereby amplifying inflammatory signaling. While IL-32 induction is beneficial for the control of pathogens (3, 4), it seems to suppress immune responses at later stages of persistent viral infection (3), suggesting an ambiguous role in immune regulation. This is further supported by conflicting reports in cancer, as increased IL-32 levels associate with enhanced anti-tumor immunity and good patient prognosis in cutaneous melanoma (5) but tumor progression in many epithelial cancers (6, 7). Taken together, these observations indicate that IL-32 is involved in the complex regulation of both immune and non-immune cells, underlining its critical role in human health and disease.

Besides the major cellular tissue components (fibroblasts, epithelial and endothelial cells), particularly immune cells contribute to IL-32 production during inflammation (2). IL-32 can be expressed by innate immune cells but in cases of viral infection, autoimmunity, and cancer the predominant IL-32 source are T cells (2, 8–10). Distinct T cell subsets play critical but complex roles during inflammation, infection and cancer immune surveillance (11). Cytotoxic CD8^+^ T cells and CD4^+^ T helper (Th) cells can eliminate pathogens and tumor cells, but their autoreactive counterparts mediate autoimmune tissue damage (11). In contrast, regulatory CD4^+^ T cells (Treg) actively suppress immune responses, preventing not only autoimmunity (12), but also the immune control of cancer (13). Despite their divergent roles, all of these T cell subsets can express *IL32* mRNA (9, 10, 14). However, first indications exist supporting that T cell subsets may express IL-32 at different levels. For example, RNA-Seq data from Peripheral Blood Mononuclear Cells (PBMCs) of healthy donors (HDs) revealed the highest *IL32* expression in immune suppressive memory Treg, followed by proinflammatory CD4^+^ effector cells (Teff), and, in particular, Th1 Teff (15). In addition, local inflammatory responses may be shaped by the differential transcription of different *IL32* isoforms (α, β, γ, δ, ϵ, η, ζ, θ, small) with distinct functions and potency (7). Thus, the degree and isoform composition of IL-32 expression in each T cell subset may contribute to its physiological role. A major body of studies has focused on IL-32γ that exerts a strong pro-inflammatory activity on various cells, including human PBMCs, monocytes and CD4^+^ T cells (3, 16, 17). Its pre-mRNA undergoes alternative splicing, generating all other IL-32 isoforms (7). The small IL-32 isoforms (α, β, δ and θ) exhibit not only various degrees of pro-inflammatory potency (17) but also context-dependent anti-inflammatory properties, including the induction of IL-10 by IL-32β (15).

Previous studies suggest that activated human T cells may predominantly transcribe the *IL32β* isoform (8, 18). However, a systematic analysis of the differential transcription and protein expression of IL-32 isoforms in T cells has not yet been conducted. Moreover, it remains unclear whether T cells actively secrete IL-32 to influence the function of other cells within the tissue microenvironment or whether IL-32 expression rather serves a primarily cell-intrinsic role. Increased levels of IL-32 in the serum of patients and in culture supernatants of various cell types that can express IL-32γ (2) support its extracellular release. In tumor cell cultures IL-32 was detected in intracellular vesicles and, thus, neighboring cells can be exposed to IL-32 through the uptake of extracellular vesicles released from IL-32 expressing cells (2, 19). Exogenous provision of human recombinant IL-32 stimulates characteristic signaling pathways and biological responses in treated cells (15–17). This suggests that the biological function of IL-32 is based at least in part on its active release and activity on neighboring cells.

Hitherto, IL-32 secretion mechanisms have not been characterized for T cells. While upon Activation Induced Cell Death (AICD) dying T cells might release IL-32 into the microenvironment (18), it remains questionable whether such process would substantially contribute to IL-32 effects. Among all isoforms, only IL-32γ but not the short IL-32 isoforms, which are expressed by T cells (8, 18), possesses an N-terminal hydrophobic signal peptide to mediate transmembrane secretion (7). Moreover, the IL-32 protein is predominantly located in the cytosol and (weakly) in the nucleus (2) and no IL-32 receptor has been identified so far (15) apart from potential unspecific IL-32 binding proteins on the cell surface, such as integrin β3 and the serine protease PR3 (2). Thus, there is overall an increased possibility that short IL-32 isoforms exert primarily cell-intrinsic functions. In this case, IL-32 produced by T cell infiltrates would not play a major role in directly orchestrating complex tissue responses to inflammation and stress.

In view of the critical role of both T cells and IL-32 in tissue homeostasis during stress and inflammation, we here focused on basic features of IL-32 induction, expression, and release by T cells. We demonstrate that activated human T cells predominantly express and secrete the IL-32β protein isoform and reveal CD4^+^ T cells as the main IL-32β producers in blood of healthy individuals, while in primary tumors of breast cancer patients Treg and Th1 Teff represent the major IL-32 producing subsets. We show that IL-32 expression in T cells is induced through IL-2 signaling and its secretion is triggered upon T Cell Receptor (TCR) stimulation. Finally, we demonstrate that T cells actively secrete IL-32 through unconventional secretion pathways, predominantly as a free protein potentially through membrane pores and secondarily through the release of exosomes.

## Materials and methods

### Healthy donors and patient samples

Peripheral blood (PB) of HDs was provided by the department of Transfusion Medicine at the University Hospital of Regensburg. PB samples were used after informed consent of all included individuals according to the certificate 21–2393-101, which was approved by the Ethics Committee of the University of Regensburg. Tumor antigen-specific cytolytic CD8^+^ T cells recognizing the survivin_(95-104)_ epitope were generated from PBMCs of HDs, as previously described (20). Myeloid infiltrating lymphocytes (MILs) were isolated from the bone marrow of a patient suffering from multiple myeloma and were expanded using the Rapid Expansion Protocol (REP), as published before (21), following the respective Ethics Committee approval of the certificate #229/2003 and S-152/2010 after written informed consent.

### Isolation of T cells from PB of HDs

PBMCs were isolated from PB using Ficoll (Pan Biotec, Cat#P04–601000)-based density-gradient centrifugation. The collected PBMCs were washed twice with Roswell Park Memorial Institute (RPMI) 1640 (Gibco, Cat#21875–034) and were incubated on tissue-treated T cell culture dishes (Stricker, Cat#93150) in X-VIVO™ 20 medium (Lonza, Cat#BE04–448Q) for 1.5 h to overnight at 37°C and 5% CO_2_, to separate suspension cells, including T cells, from adherent monocytes. Non-adherent cells were carefully collected and washed with RPMI 1640 followed by centrifugation for 10 min at 500xg at RT. Finally, T-cell-containing non-adherent cells were cultured in T cell medium, which consists of X-VIVO™ 20 supplemented with 100 IU/ml IL-2 (IL-2 Proleukin® S, Novartis Pharma, Cat#1735) and 60 IU/ml IL-4 (Miltenyi Biotec, Cat#130–093-922) for 1–7 days. Subsequently, CD3^+^ T cells were isolated from the cultures of non-adherent cells using the Dynabeads™ Untouched™ Human T Cells Kit (Thermo Fisher Scientific, Cat#11344D) according to the manufacturer’s instructions.

### Cell culture of T cells

Survivin-specific T cells and MILs were cultured in complete lymphocyte medium (CLM), namely RPMI 1640 (Gibco, Cat#21875–034) containing 10% human albumin (hAB) serum (Valley Biomedical, Cat#HP1022), 1% Penicillin-Streptomycin (P/S; Sigma-Aldrich, Cat#P4333), 1% HEPES (Sigma-Aldrich, Cat#H0887) and 0.01% β-Mercaptoethanol (Gibco, Cat#31350–010) in cell culture flasks (Greiner Bio-one, Cat#690175, 658175, 660175) at a cell density of 1x10^6^ and 0.6x10^6^ cells/ml, respectively. HD CD3^+^, CD4^+^, and CD8^+^ T cells were cultured at 1x10^6^ cells/ml of T cell medium in cell culture dishes (Stricker, Cat#93100, 93150). Every two to three days, half of the culture supernatant was exchanged with fresh medium.

### Polyclonal stimulation of T cells

T cells were activated using non-tissue treated FB culture plates (12-well, Falcon, Cat#351143; 24-well, Falcon, Cat#351147; 96-well, Falcon, Cat#351172; 48-well, Thermo Fisher Scientific, Cat#152640), which had been coated overnight at 4°C with 1X PBS (Sigma, Cat#D1408) containing 4 µg/ml anti-CD3 antibodies (eBioscience, Cat#14–0037-82, RRID: AB_467057). After washing the coated wells/plates with PBS (Sigma, Cat#D1408), the T cells were seeded at a density of 1x10^6^ cells/ml of culture medium (CLM: Survivin T cells and MILs, T cell medium: HD T cells) supplemented with 1 µg/ml anti-CD28 antibody (BioLegend, Cat#302902, RRID: AB_314304). T cells were incubated for defined time periods at 37°C and 5% CO_2_. To investigate the impact of IFN-γ and IL-2 on IL-32 intracellular expression and extracellular secretion, T cells were cultured for 24 h at 37°C in the presence of either 50 ng/ml recombinant human IFN-γ (PeproTech, Cat#300–02-100UG) or 100 IU/ml IL-2 (IL-2 Proleukin® S, Novartis Pharma, Cat#1735) for low IL-2 treatment and 3,000 IU/ml IL-2 for high IL-2 treatment without polyclonal stimulation. As a negative control, T cells were cultured without stimulation, while polyclonal stimulation without IFN-γ or IL-2 was used as a positive control. CD25 signaling was inhibited by IL-2 ligand blocking using Human anti-IL-2 antibodies (R&D Systems, Cat#MAB202R-SP, RRID: NA, 1.5 µg/ml), while Mouse IgG1 Isotype Control (R&D Systems, Cat#MAB002, RRID: AB_357344, 1.5 µg/ml) was used as negative control. During IL-2 ligand blocking the medium was additionally supplemented with recombinant BenzNuclease/Benzonase Protein (Speed BioSystems, YCP1200–500KU, 600 U/ml) to prevent the clustering of T cells upon polyclonal stimulation. This facilitated the binding of anti-IL-2 antibodies to soluble IL-2 secreted by activated T cells before the ligation of IL-2 on CD25 on the surface of activated T cells.

### Inhibition of IL-32 secretion by Survivin-specific T cells

Survivin-specific T cells were cultured at a density of 0.6x10^6^ cells in 600 µl CLM per well in a 48-well non-tissue treated FB culture plate (Thermo Fisher Scientific, Cat#152640) for 4 h without or with polyclonal stimulation either alone or in the presence of the secretion inhibitors Monensin, Brefeldin A (BFA), NH_4_Cl or Punicalagin. Monensin (4 µl/6 ml; BD Biosciences, Cat#554724), Brefeldin A (BFA; 1 µl/ml; BD Biosciences, Cat#555029) and NH_4_Cl (50 mM; Roth, Cat#K298.1) were supplemented at the beginning of the polyclonal stimulation, while Punicalagin (2.5, 5, or 50 µM; Sigma-Aldrich, Cat#P0023–1MG) was already added to Survivin T cells 10 min before stimulation. Since the diluent of Punicalagin is 99.9% methanol (MeOH; Analytical reagent grade; Fisher Chemical, Cat#M/4000/17), we also included for each applied Punicalagin concentration, the respective volume of methanol, as a negative control.

### Flow cytometry

Protein expression at the single-cell level was analyzed by flow cytometry (FC) for markers expressed on the plasma membrane or intracellular flow cytometry (ICFC) for markers localized in the nucleus and/or the cytoplasm. First, to inhibit unspecific binding of antibodies on Fc receptors on the cell surface, T cells were incubated for 15 min on ice with human Ig (Kiovig, Baxter, Cat#PZN-06587176) diluted 1:20 in FACS buffer, namely PBS (Sigma, Cat#D1408) containing 2% fetal calf serum (FCS; Sigma-Aldrich, Cat#F7524). To exclude dead cells, after washing with PBS (Sigma, Cat#D1408), the cells were stained with the Zombie NIR™ Fixable Viability Kit (BioLegend, Cat#423105) or the Zombie Aqua™ Fixable Viability Kit (BioLegend, Cat#423102) according to the manufacturer’s recommendations. To analyze CD25 expression, cells were first washed with MACS buffer, which contains 2 mM EDTA (Invitrogen, Cat#15575–038) and 0.5% hAB serum in PBS (Sigma, Cat#D1408), and then incubated with anti-CD25-VioBright-FITC (Miltenyi Biotec, Cat#130–113-283, RRID: AB_2734062, 1:50) or the mIgG2b,κ-VioBright-FITC isotype control (Miltenyi Biotec, Cat#130–104-649, RRID: AB_2661748, 1:9) in MACS buffer for 10 min at 4–8°C. After washing with FACS buffer, the cells were incubated with anti-CD3-BV605 (BioLegend, Cat#300459, RRID: AB_2564379, 1:20) and anti-CD4-PerCP (BioLegend, Cat#300528, RRID: AB_893321, 1:20) in FACS buffer for 20 min on ice. For intracellular IL-32 expression analysis, the cells were first washed with FACS buffer and then fixed/permeabilized using either the eBioscience™ Foxp3/Transcription Factor Fixation/Permeabilization Concentrate and Diluent (eBioscience, Cat# 00–5521-00) or the BD Pharmingen™ Transcription Factor Buffer Set (BD Biosciences, Cat# 562574). According to the eBioscience protocol, the cells were first fixed in a mixture of concentrate and diluent at a ratio of 1:3 for 30 min on ice and then permeabilized with Perm buffer for 20 min on ice. Following the BD Biosciences protocol, the cells were fixed and permeabilized using the Fix/Perm buffer (Concentrate diluted 1:4 in Transcription Factor Diluent) for 50 min at 4°C. For both protocols, the cells were then washed twice with the respective Perm buffer and incubated with mouse serum (ThermoFisher Scientific, Cat#24–5544-94) diluted 1:100 in Perm buffer for 30 min at RT. After washing with Perm buffer, the cells were stained with anti-IL-32αβγδ-Pacific Blue™ (BioLegend, Cat#513501, RRID: AB_2124018, after customized labeling, 1:25–56) or mIgG1,κ-Pacific Blue™ isotype control (BioLegend, Cat#400131, RRID: AB_2923473, 1:25–52) in Perm buffer for 30 min at RT. After the last washing step, the cells were resuspended in FACS buffer and analyzed in a BD FACSLyric with FACSuite software (BD Bioscience, v1.2.1.5657). The acquired data were analyzed using FlowJo (BD Bioscience, v10.8.1, RRID: SCR_008520). In addition, we included ‘Fluorescent Minus One (FMO) + isotype’ controls containing antibodies against all markers except one that is replaced by the respective isotype control.

### CRISPR/Cas9-genome editing of Survivin-specific T cells

Genome editing of Survivin-specific T cells was performed using the Alt-R® CRISPR-Cas9 System [Integrated DNA Technologies (IDT)] after adapting a previously published protocol (22). First, to prepare the IL-32-specific and the Scr negative control guide RNA (gRNA), the CRISPR-RNA Alt-R® CRISPR-Cas9 crRNA Hs.Cas9.IL32.1.AB or the Alt-R® CRISPR-Cas9 Negative Control crRNA #1, respectively, was mixed with the trans-activating crRNA Alt-R® S.p. HiFi Cas9 tracrRNA (IDT, Cat#1072534) at a 1:1 ratio in a final volume of 3 µl to reach a final concentration of 50 µM. The crRNA-tracrRNA mix was denatured for 5 min at 95°C followed by 10 min at RT. The obtained gRNA was then mixed with the Alt-R® S.p. HiFi Cas9 Nuclease V3 (IDT, Cat#1081061) at a ratio of 3:1.2 and incubated for 20 min at RT to form a ribonucleoprotein (RNP) complex. The RNP complex was combined with a maximum of 10^6^ Survivin-specific T cells in 20 µl of P3 buffer containing Supplement at 1:5.55 dilution (P3 Primary Cell 4D Nucleofector X Kit S, Lonza, Cat#V4XP-3032). The RNP-cell suspension was then transferred to 16-well Nucleocuvette™ Strips and the pulse code EH-100 was applied using the Amaxa 4D-Nucleofector® X Unit (Lonza). Finally, the nucleofected cells were supplemented with 70 µl of pre-warmed X-VIVO™ 20 medium containing 100 IU/ml IL-2 (IL-2 Proleukin® S, Novartis Pharma, Cat#1735) and incubated for at least 15 min at 37°C and 5% CO_2_. The cells were then transferred into cell culture plates and incubated in X-VIVO™ 20 medium with 100 IU/ml IL-2 for 3 days, followed by 2 days of treatment with 3,000 IU/ml IL-2 (IL-2 Proleukin® S, Novartis Pharma, Cat#1735) at a cell density of 10^6^ cells/ml. Afterwards, the knock-out (KO) efficiency was determined by ICFC analysis, while the remaining cells were further cultivated in CLM without IL-2 overnight. The next day, Scr and IL-32 KO Survivin-specific T cells underwent a rapid expansion protocol (REP), as previously described (23). In brief, nucleofected cells were co-cultured with irradiated feeder cells, which contained PBMCs from three HDs, at a ratio of 1:100 in the presence of 30 ng/ml anti-CD3 antibodies (eBioscience, Cat#14–0037-82, RRID: AB_467057) and 3,000 IU/ml IL-2 (IL-2 Proleukin® S, Novartis Pharma, Cat#1735) in medium, including CLM and AIM-V (Thermo Fisher Scientific, 12055091) at 1:1 ratio. After 5 days, the culture medium was refreshed with 3,000 IU/ml IL-2 (IL-2 Proleukin® S, Novartis Pharma, Cat#1735). Between day 7 and day 14 of the expansion, 2/3 of the culture medium were refreshed every 2–3 days and the cell number was counted to sustain a cell density of 0.6x10^6^ cells/ml. Finally, on day 14 the IL-32 KO efficiency was validated using ICFC and cell aliquots were stored in freezing medium containing 20% RPMI 1640 (Gibco, Cat#21875–034), 70% FCS (Sigma-Aldrich, Cat#F7524) and 10% DMSO (Sigma-Aldrich, Cat#D2650) in liquid nitrogen.

### Extracellular vesicle isolation

Extracellular vesicles (EVs) and exosomes were isolated from Survivin-specific T cell cultures using an adaptation of a previously published protocol (24). In detail, 5x10^7^ Survivin T cells were polyclonally stimulated for 4 h in 50 ml of EV-free X-VIVO 20 medium, which was prepared after ultracentrifugation of X-VIVO™ 20 (Lonza, Cat#BE04–448Q) at 100,000xg for 18 h at 4°C using the Ultracentrifuge Optima XE-90 (Beckman Coulter Life Sciences, Cat#A94471). The resulting cell solution, which contained both Survivin T cells and the culture supernatant, was divided in 2 parts with 5 ml being stored in ice for protein enrichment, as described in detail below, and the remaining 45 ml being centrifuged for 5 min at 500xg at 4°C for cell depletion. The cell pellet was washed from contaminating supernatant proteins with PBS (Sigma, Cat#D1408) and after centrifugation for 5 min at 500xg at 4°C was resuspended in 450 µl of lysis buffer containing phosphatase and proteinase inhibitors for protein lysate preparation, as described in detail below. The cell-free supernatant was in part (5 ml) stored in ice for protein enrichment, while the remaining 40 ml were further centrifuged for 10 min at 1,000xg at 4°C to deplete cell debris. The cell debris pellet was resuspended in PBS (Sigma, Cat#D1408), centrifuged for 10 min at 1,000xg at 4°C and resuspended in 40 µl of lysis buffer. A sample (5 ml) of the cell debris-free supernatant was stored in ice for protein enrichment and the remaining 35 ml were centrifuged for 30 min at 10,000xg at 4°C to deplete microvesicles. The microvesicle pellet was washed with PBS, centrifuged for 30 min at 10,000xg at 4°C and resuspended in 35 µl of lysis buffer. The microvesicle-free supernatant was stored in part (5 ml) in ice for protein enrichment and the remaining 30 ml were ultracentrifuged for 18 h at 100,000xg at 4°C to deplete exosomes. The exosome-free supernatant (5 ml) was used for protein enrichment analysis. The exosome pellet was washed with PBS, ultracentrifuged for 1.5 h at 100,000xg at 4°C and resuspended in 30 µl of lysis buffer. Finally, 5 ml of the PBS-wash supernatant but also 5 ml of the EV-free X-VIVO 20 medium also underwent protein enrichment.

### RNA isolation, reverse transcription and quantitative RT-qPCR

Total RNA was isolated from cell pellets using the RNeasy® Mini Kit (Qiagen, Cat#74106) or the RNeasy® Micro Kit (Qiagen, Cat#74004), in case of cell numbers lower than 5x10^5^, following the manufacturer’s instructions. RNA was eluted in molecular grade RNAse-free water and its concentration and quality were spectrophotometrically analyzed using the NanoDrop™ 2000c (Thermo Fisher Scientific). Total RNA, maximum 1 µg per reaction, was reversely transcribed into cDNA using the QuantiTect® Reverse Transcription Kit (Qiagen, Cat#205313) according to the manufacturer’s protocol. The resulting cDNA samples were diluted in molecular grade RNase-free water in preparation for gene expression analysis using the QuantiFast® SYBR® Green PCR Kit (Qiagen, Cat#204057) and, after its discontinuation, the QuantiNova® SYBR® Green PCR Kit (Qiagen, Cat#208056). For each reaction, 2 µl of cDNA (undiluted/diluted in molecular grade RNAse-free water), 10 µl of the 2X QuantiFast® SYBR® Green PCR mix or the QuantiNova® SYBR® Green PCR Master Mix combined with QN ROX Reference Dye (1:100) and 0.4 µM of the respective forward and reverse primer (**Supplementary Table 1**) were mixed to a total volume of 20 µl. To this end, we used *IL32* isoform-specific primer pairs that were designed to detect the main *IL32* isoforms *IL32α (A)*, *IL32β (B)*, *IL32γ (E)*, *IL32η (C)*, and *IL32D* and had been already validated before (25). In addition, we included a primer pair that binds a common sequence fraction of all *IL32* isoforms (26) to assess the total IL32 mRNA expression. Each sample was analyzed in triplicates and a negative control without template cDNA was included per mastermix. All reactions were pipetted into MicroAmp™ Optical 96-Well Reaction Plates (Thermo Fisher Scientific, Cat#N8010560) covered by MicroAmp™ Optical Adhesive Films (Thermo Fisher Scientific, Cat#4311971). The RT-qPCR reaction program (**Supplementary Table 2**) was run in the QuantStudio™ 3 Real-Time PCR system (Life Technologies). The analysis was performed using the comparative Ct method (ΔΔCt). Target gene expression levels were normalized to the expression levels of the housekeeping gene *PPIB* or *ACTB*.

### Enzyme-linked immunosorbent assay (ELISA)

The concentration of IL-32 and TNF-α in culture supernatants was measured using the Human IL-32 DuoSet ELISA (R&D Systems, Cat#DY3040–05) and the Human IFN-γ ELISA Set (BD Biosciences, Cat#555142), respectively, according to the manufacturer’s instructions. Colorimetric substrate reactions by horseradish peroxide (HRP) were measured at λ = 450 nm with λ = 570 nm as a reference wavelength using the multimode microplate reader Spark 10M (Tecan). Each sample was analyzed in duplicates or triplicates and negative controls (cell culture medium only) were included in every run. Negatively calculated values are displayed as zero/non-detectable values.

### Luminex assay

The concentration of IL-2 in T cell culture supernatants was determined using the MILLIPLEX Human CD8^+^ T Cell MAGNETIC Premixed 17 Plex Kit (Merck, Cat#HCD8MAG15K17PMX) following the manufacturer’s recommendations. Measurements were performed using the MAGPIX Luminex instrument (Merck).

### Protein lysate preparation from cells, cell debris, microvesicles and exosomes

The harvested pellets were washed with ice-cold PBS and were either snap-frozen in liquid nitrogen and stored at -80°C or lysed directly in 30 to 50 µl of Cell Signaling Lysis Buffer (Merck Millipore, Cat#43–040) supplemented with protease and phosphatase inhibitors (Protease Inhibitor Cocktail Set III, EDTA-Free, Merck Millipore, Cat#539134–1ML, 1:100 or cOmplete™, EDTA-free Protease Inhibitor Cocktail, Roche, Merck Millipore, Cat#04693132001, 1:4; Phosphatase Inhibitor Cocktail 3, Merck Millipore, Cat#P0044–1ML, 1:100) for 15 min in the fridge using a MACSmix^TM^ Tube Rotator (Miltenyi Biotec). Last, after centrifugation for 15 min at 17,000xg at 4°C, the protein-containing supernatants were retained as cell lysates.

### Protein enrichment from T cell culture supernatants

Proteins in the supernatant of T cell cultures were enriched using either Amicon Ultra Centrifugal Filters [Merck Millipore, Cat#UFC801024, 10 kDa molecular weight cut-off (MWCO)] or trichloroacetic acid (Merck Millipore, Cat#1.00807.0250)/acetone (Roth, Cat#32201) (TCA/Ac) precipitation. Irrespective of the enrichment method, the supernatants were supplemented with both protease and phosphatase inhibitors, the same as the ones used for protein lysate preparation but in a 10-fold higher dilution. Using Amicon Ultra Centrifugal Filters, the supernatant was concentrated 20-fold after centrifugation for 15 min at 4,000xg in a swinging-bucket rotor. Protein enrichment using the TCA/Ac precipitation was performed based on an adapted published protocol (27). In brief, culture supernatants were incubated with TCA at a ratio of 1:10 for 30 min on ice. The released proteins were pelleted for 15 min at 10,000xg at 4°C and washed two times with ice-cold acetone. Finally, the air-dried pellets were resuspended in 25 μl of molecular grade water or, in the case of EV isolation, in 50 µl of Cell Signaling Lysis Buffer, as described for protein lysate preparation.

### Western Blot analysis

Protein concentration was determined using the Pierce™ BCA Protein Assay Kit (Thermo Fisher Scientific, Cat#23225) following the manufacturer’s instructions. 10–30 µg protein per cell lysate or enriched supernatant were incubated with NuPAGE™ LDS Sample buffer (Thermo Fisher Scientific, Cat#NP0007) containing 10% β-Mercaptoethanol (Roth, Cat#4227.1) for 10 min at 70°C in the ThermoMixer® C (Eppendorf). The samples were then loaded on Novex™ NuPAGE™ 4–12% Bis-Tris Protein-Gels (Thermo Fisher Scientific, Cat#NP0321BOX, NP0336BOX) in Mini Gel Tanks (Thermo Fisher Scientific) in the presence of NuPAGE™ MOPS SDS Running Buffer (Thermo Fisher Scientific, Cat#NP0001). To identify IL-32 isoforms, 4 – 6 ng of human recombinant IL-32β (rIL-32β, R&D Systems, Cat#6769-IL-025) and/or human recombinant IL-32γ (rIL-32γ, R&D Systems, Cat#4690-IL-025/CF) were loaded next to the samples, while 6 µl of the PageRuler™ Prestained Protein Ladder (Thermo Fisher Scientific, Cat#26616) were included to identify the MW of the analyzed proteins. The proteins were separated using the PowerEase® 300W Power supply (Thermo Fisher Scientific) and were blotted using the Trans-Blot® Turbo™ RTA Mini 0.45 µm Nitrocellulose Transfer Kit (Bio-Rad, Cat#1620115) according to the manufacturer’s instructions. The transfer was performed at the Trans-Blot® Turbo™ Transfer System using the “High MW” program of Bio-Rad at 25 V extended to 30 min. Transfer efficiency was determined by staining the remaining proteins in the gel with Bio-Safe™ Coomassie G-250 Stain (Bio-Rad, Cat#161–0786) and the transferred proteins on the membrane with Ponceau S solution (Sigma-Aldrich, Cat#p7170–1l) following the manufacturer’s instructions. After the Ponceau S staining, the membrane was first cleaned using TBS-T, namely 0.05% Tween-20 (AppliChem, Cat#A4974,0250) in TBS, which contains 15 mM Tris(hydroxymethyl)aminomethane (Merck Millipore, Cat#1.08382.0500) and 10 mM Sodium chloride (VWR, Cat#27810.364) in ddH_2_O. The membrane was blocked with 10% milk (Powdered milk, Roth, Cat#T145.1)-TBS-T solution (IL-32) or 1X animal-free blocking solution (AFBS, Cell Signalling, Cat#15019L; remaining proteins) for 2 h at RT on a Sunlab® 3D-Shaker (Sunlab). After two washes with 1% milk-TBS-T (IL-32) or TBS-T (remaining proteins) for 5 min, the membrane was incubated overnight at 4°C on the Assistent RM 5 (Ingenieurbüro CAT M. Zipperer GmbH) under continuous rotation with 3–5 ml of 1% milk-TBS-T containing the anti-IL-32 antibodies (Purified anti-human IL-32αβγδ, BioLegend, Cat#513501, RRID: AB_2124018, 1:500), or 1X AFBS containing anti-Grp94 (Enzo Life ScienceS, Cat#ADI-SPA-850-D, RRID: AB_2039133; 1:1,000) and anti-Hsp70 (BD Biosciences, Cat#610607, RRID: AB_397941, 1:1,000) or anti-GAPDH (Santa Cruz Biotechnology, Cat#sc-47724, RRID: AB_627678, 1:1,000) or anti-pSTAT5 (Tyr694) (Cell Signaling, Cat#9359S, RRID: NA, 1:1,000). The membranes were then washed three times with either 1% milk-TBS-T (IL-32) or TBS-T (remaining proteins) for 10 min. Subsequently the membrane was incubated with AFBS containing HPR-coupled anti-mouse IgG (Cell Signalling, Cat#7076S, RRID: NA, 1:4,000; IL-32, Hsp70, GAPDH), HPR-coupled anti-rat IgG (Sigma, Cat#7077, 1:3,000, Gpr94) or HPR-coupled anti-rabbit IgG (Cell Signalling, Cat#7074S, RRID: NA, 1:2,000, pSTAT5 (Tyr694)) secondary antibody for 1 h at RT. After three washing steps with 1% milk-TBS-T, TBS-T and finally TBS (IL-32) or TBS-T two times and finally TBS (remaining proteins) for 10 min, the membrane was incubated with a mix of the two substrates at a ratio of 1:1 using either the Pierce™ ECL Western Blotting Substrate (GAPDH; Thermo Fisher Scientific, Cat#32209) or Trident femto Western HRP Substrate (remaining proteins; GeneTex, Cat#GTX14698) for 1 min (GAPDH) or 5 min (remaining proteins). Finally, the ChemiDoc™ Imaging System (Bio-Rad) was used to acquire both colorimetric and chemiluminescent blot images. Protein levels were quantified using ImageJ (Wayane Rasband, v1.53t, RRID: SCR_003070). Background noise was removed by defining a closed area under the curve (AUC) for the protein-specific peak. Intracellular protein expression levels were normalized to the GAPDH values.

### *IL32*-isoform identification

*A*ll available Ensembl *IL32* transcripts (IL-32–201 – IL-32–235) were assigned to specific *IL32* isoforms based on their exon-intron usage profile and the Greek nomenclature according to Sloot et al. (7) together with the NCBI nomenclature. The tool Kallisto (28, https://pachterlab.github.io/kallisto/) was then applied as part of the TraCeR workflow (29, https://github.com/Teichlab/tracer) to determine the presence of each Ensembl *IL32* transcript ID as Transcripts Per Million (TPM) per single-cell transcriptome of all cells included per cluster in a previously published single-cell transcriptome dataset (14). Finally, TPM values that correspond to Ensembl transcript IDs of the same isoform (e.g. *IL32β*) were added together.

### Statistical analysis

Statistical analysis of differences between compared groups was performed using GraphPad Prism 9 (Dotmatics). Two-sided Student’s unpaired t-test was used to compare values from duplicates or triplicates within each experiment and two-sided Student’s paired t-test for cumulative data of at least n=3 individual experiments using their mean values. Significances were denoted as *p<0.05, **p<0.01, ***p<0.001 and ****p<0.0001.

## Results

### T cells predominantly express the IL-32β isoform in healthy individuals and breast cancer patients

We first studied *IL32* isoform expression on the cellular level in human CD4^+^ Th subsets. To this end, we analyzed a previously published data set containing single-cell transcriptomes of CD4^+^ T cells isolated from primary human breast tumors and the PB from the corresponding patients (14). These included tumor-infiltrating early activated Tconv, uncommitted Teff, Th1 Teff, suppressive Treg and protumorigenic Treg but also circulating breast tumor antigen reactive Th1 Teff. Importantly, single-cell transcriptomes were generated using Smart-Seq2 for single-cell poly(A) RNA amplification, which facilitates full length mRNA characterization (30), and deep sequencing at a high coverage of ~1.3 million reads per single cell (14), allowing the efficient quantification of different *IL32* isoform transcripts.

**Figure 1:**
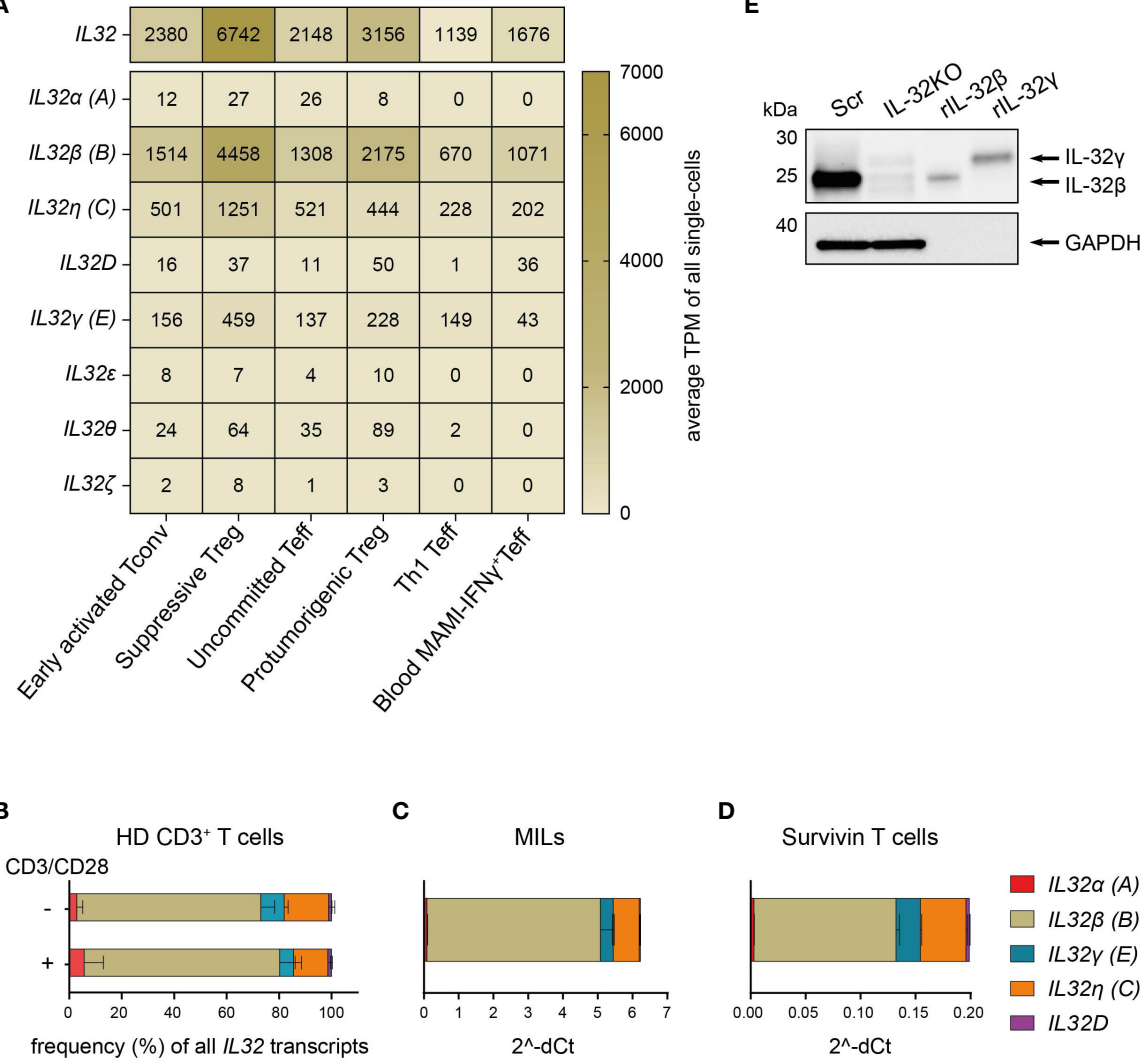
T cells predominantly express the IL-32β isoform. **(A)** Expression distribution of total *IL32* and the *IL32* isoforms α **(A)**, β **(B)**, η **(C)**, D, γ **(E)**, ϵ, θ, and ζ in the functional transcriptome clusters of Early activated Tconv, Suppressive Treg, Uncommitted Teff, Th1 Teff and Protumorigenic Treg among intratumoral CD4^+^ T cells (n=4) or in circulating mammaglobulin (MAMI)-reactive IFNγ^+^CD4^+^Teff (n=3) of breast cancer patients depicted as average Transcripts Per Million (TPM) of all single-cells included in each cluster and based on previously published single-cell transcriptome sequencing data (14). **(B–D)** Relative expression of the *IL32* isoforms α **(A)**, β **(B)**, γ **(E)**, η **(C)** and D among **(B)** T cells isolated from peripheral blood of healthy donors (HDs, n=4) either after stimulation with anti-CD3/CD28 antibodies (+) or after resting (-) for 24 h, **(C)** myeloid infiltrating lymphocytes (MILs) isolated from a multiple myeloma patient and **(D)** Survivin-specific T cells measured by RT-qPCR analyses. **(B)** The proportion (%) of each isoform among all detected transcripts of the tested *IL32* isoforms. **(C, D)** Ct value of each tested *IL32* isoform normalized to the Ct value of the housekeeping gene **(C)** *PPIB* and **(B, D)** *ACTB* (2^-ΔCt). Mean+SD. MILs: n=2, Survivin T cells n=2, n corresponds to independent experiments. **(E)** WB analysis of IL-32 expression in cell lysates (27 µg) of Scr and IL-32KO Survivin T cells next to rIL-32β (4 ng, MW: 23.1 kDa) and rIL-32γ (4 ng, MW: 28.1 kDa). As a loading control, the housekeeping gene GAPDH (37 kDa) was detected on the same blot. Representative blot of n=6 independent experiments. MW, Molecular Weight.

First, we grouped all available Ensembl *IL32* transcripts (IL-32–201 – IL-32–235) based on their similarities in exon-intron patterns and named them using the Greek nomenclature according to Sloot *et al*. (7) in combination with the NCBI nomenclature. We then performed a distribution analysis of the assigned *IL32* isoform-specific sequences in the single-cell RNA sequencing (scRNASeq) data from breast cancer patients. *IL32* transcripts were present in all intratumoral Th subsets, though at different levels, with the highest expression in suppressive Treg followed by Th1 Teff, but also in circulating IFNγ^+^CD4^+^ Teff (Th1) reactive to the breast tumor antigen mammaglobulin (MAMI) (**Figure 1A**). *IL32β* was clearly the predominant isoform in all studied subsets followed by *IL32η* and *IL32γ* (**Figure 1A**). We also quantified total *IL32* and *IL32* isoform-specific transcripts in resting or polyclonally activated CD3^+^ T cells isolated from PB of HDs by isoform-specific RT-qPCR analyses. In agreement with tumor-infiltrating T cells, both resting and polyclonally stimulated CD3^+^ T cells of HDs transcribed predominantly the *IL32β* isoform followed by *IL32η* and *IL32γ*, while *IL32α* and *IL32D* were barely detectable (**Figure 1B**). The same *IL32* isoform distribution was displayed in two sources of tumor antigen (TA)-specific T cells, namely CD4^+^ and CD8^+^ MILs (21) and CD8^+^ Survivin-specific T cells (20, **Figures 1C, D**).

Western Blot (WB) analyses of cell lysates from Survivin-specific T cells compared to the respective IL-32 knockout (IL-32KO) variant demonstrated that T cells mainly express the IL-32β isoform on the protein level (**Figure 1E** and **Supplementary Figures 1A, B**). This was based on the fact that the molecular weight (MW) of IL-32 derived from the Scr control of Survivin-specific T cells resembled that of the human recombinant protein IL-32β (rIL-32β) and was smaller than that of rIL-32γ, while the respective band was barely detectable in IL-32KO cells. Taken together, IL-32β is the prevailing isoform of IL-32 both on the mRNA and the protein level in various T cell subsets not only in healthy individuals but also in cancer patients. Moreover, among tumor infiltrating CD4^+^ T cell subsets *IL32β* is expressed at highest amounts by suppressive Treg and Th1 Teff.

### T cells upregulate IL-32 after TCR stimulation

**Figure 2:**
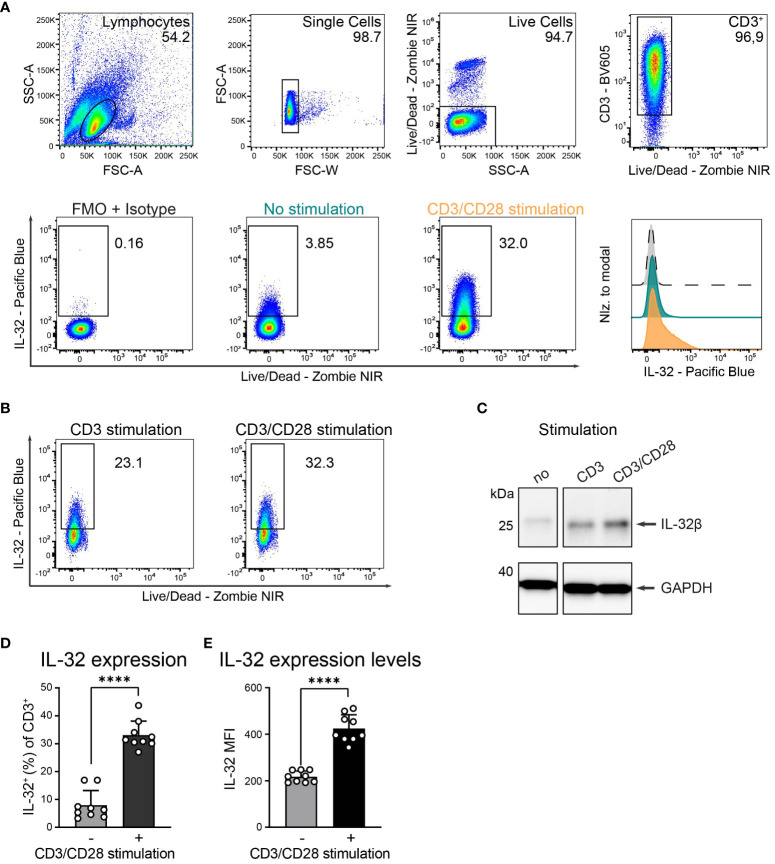
HD T cells upregulate IL-32 after TCR stimulation. **(A)** IL-32 expression in T cells isolated from peripheral blood of Healthy Donors (HDs) based on ICFC analysis. (Top) Representative gating strategy applied to identify live single CD3^+^ T cells in cultures that received 24 h of CD3/CD28 stimulation. Representative (bottom, left) dot plots of IL-32^+^ cells among CD3^+^ T cells after 24 h of resting (no stimulation, green) or CD3/CD28 stimulation (orange) and (bottom, right) histograms of IL-32 expression on CD3^+^ T cells normalized to modal based on the respective fluorescence minus one (FMO) control supplemented with the isotype control corresponding to the anti-IL-32 antibody (grey). **(B)** Representative dot plots of IL-32 expression of HD T cells after CD3 versus CD3/CD28 stimulation (n=2). **(C)** Representative WB analysis of IL-32 expression in cell lysates from unstimulated HD T cells compared to stimulation with anti-CD3 or anti-CD3/CD28 antibodies. As a loading control, the housekeeping gene GAPDH was detected on the same blot (n=2). **(D, E)** Cumulative data of **(D)** the frequency of IL-32^+^ cells or **(E)** the Mean Fluorescence Intensity (MFI) of IL-32 among live single CD3^+^ T cells without or with CD3/CD28 stimulation, n=9, mean+SD, data from individual experiments are represented as dots, paired Student’s t-test, ****p<0.0001.

Cytokine expression and secretion in T cells are commonly dependent on TCR stimulation and are further enhanced by CD28 co-stimulation (31). We, therefore, investigated the role of polyclonal T cell activation on IL-32 protein expression by intracellular flow cytometry (ICFC) analysis of blood-derived T cells from HDs. TCR stimulation resulted in strong IL-32 upregulation (**Figures 2A–C** and **Supplementary Figures 2A, B**). This was further enhanced by additional activation of the costimulatory receptor CD28 (**Figures 2B, C** and **Supplementary Figures 2A, B**). Cumulative data from nine HDs demonstrated that only a minority of unstimulated T cells expressed IL-32, ranging from 3.2% to 16.9% with a mean frequency of 7.9% (**Figures 2A, D**). However, TCR stimulation combined with CD28 costimulation triggered IL-32 expression on a large fraction (27% to 43.7%, mean: 33.1%) of the T cell population (**Figures 2A, D**). Furthermore, the mean fluorescence intensity (MFI) of IL-32 expression increased significantly after polyclonal activation compared to resting conditions (**Figures 2A, E**, MFI: 425 and 217, respectively).

**Figure 3:**
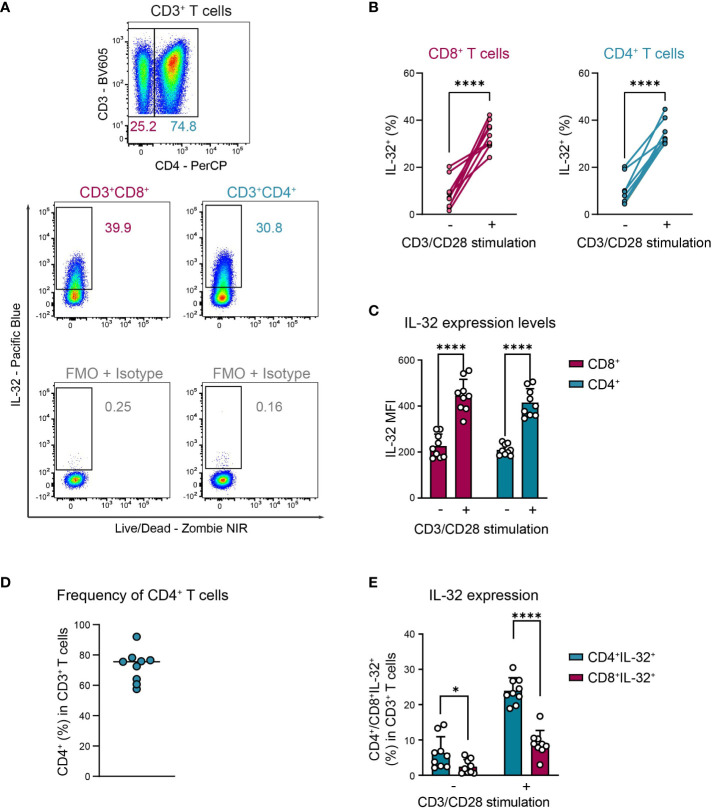
CD4^+^ and CD8^+^ T cells upregulate IL-32 after TCR activation similarly. ICFC analyses of IL-32 expression among CD4^+^ and CD8^+^ CD3^+^ T cells from blood of HDs (n=9) after 24 h of resting or CD3/CD28 stimulation. **(A)** Representative gating strategy used to identify (top) CD4^+^ and CD8^+^ (CD4^-^) cells among live single CD3^+^ T cells after 24 h of CD3/CD28 stimulation. Further identification (bottom) of IL-32^+^ cells among CD4^+^ and CD8^+^ CD3^+^ T cells according to the respective FMO + Isotype control. **(B, C)** Cumulative data of **(B)** the frequency of IL-32^+^ cells (%) and **(C)** the expression level of IL-32 per single-cell displayed by the Mean Fluorescence Intensity (MFI) among CD8^+^ and CD4^+^ CD3^+^ T cells after 24 h without or with CD3/CD28 stimulation, data from individual experiments are represented as dots, mean+SD, Student’s paired t-test, ****p<0.0001. **(D)** Frequency of CD4^+^ T cells (%) among live single CD3^+^ T cells after 24 h of CD3/CD28 stimulation, median with individual values. **(E)** Frequency of CD4^+^IL-32^+^ or CD8^+^IL-32^+^ T cells (%) among all live single CD3^+^ T cells after 24 h without or with CD3/CD28 stimulation. Mean+SD, dots depict data from individual experiments, Student’s paired t-test, *p<0.05, ****p<0.0001.

Activation-induced IL-32 upregulation was comparable between CD4^+^ and CD8^+^ T cells, regarding both the proportion of IL-32 expressing cells (**Figures 3A, B**, CD8^+^: 9% to 34%, CD4^+^: 9% to 33%) and the expression levels of IL-32 (**Figure 3C**, CD8^+^ MFI: 226 to 445, CD4^+^ MFI: 210 to 415). Still, CD4^+^ cells represented the major IL-32 expressing T cell population in the studied cohort, as their frequency among all CD3^+^ T cells was on average 72% and, thus, much higher than the proportion of the CD8^+^ subset (**Figures 3D, E**). Taken together, these data demonstrate that both CD4^+^ and CD8^+^ T cells from healthy individuals upregulate intracellular IL-32 expression after TCR stimulation.

**Figure 4:**
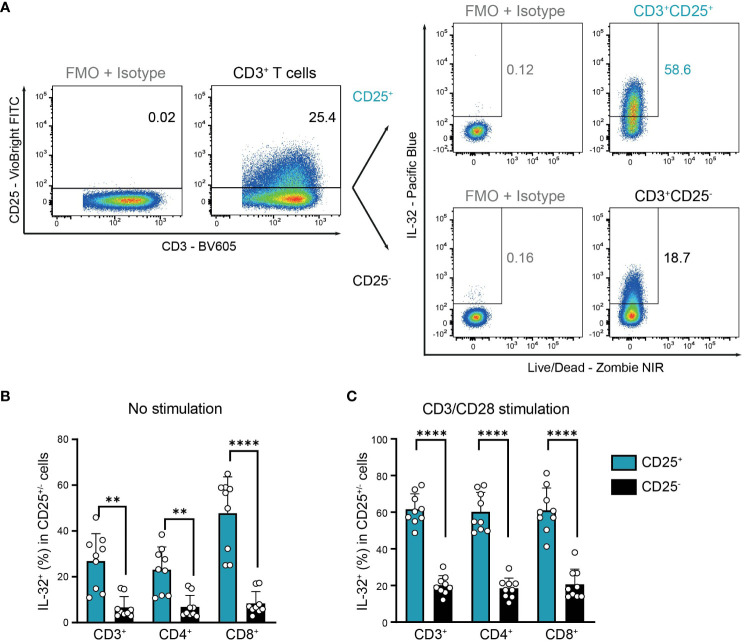
Co-expression of IL-32 and CD25 by HD T cells. IL-32 expression in CD25^+^ compared to CD25^-^ CD3^+^ T cells from healthy donors (HDs) after 24 h of resting (no stimulation) or CD3/CD28 stimulation determined by ICFC analysis. **(A)** Representative gating of CD25^+^ and CD25^-^ cells among CD3/CD28 stimulated live single CD3^+^ T cells followed by further subgating of IL-32^+^ cells among CD25^+^ or CD25^-^ cells. Gates were set according to the respective FMO + Isotype controls for CD25 and IL-32. **(B, C)** Frequency of IL-32^+^ cells (%) in CD25^+^ and CD25^-^ cells among CD3^+^, CD4^+^, and CD8^+^ T cells after 24 h **(B)** without stimulation or **(C)** with CD3/CD28 stimulation. Cumulative data of n=9, Mean+SD, dots depict data from individual experiments, Student’s paired t-test, **p<0.01, ****p<0.0001.

This suggests that IL-32^+^ cells in resting T cell populations may correspond to previously activated antigen-experienced T cells that are characterized by the expression of CD25, the high-affinity IL-2 receptor α-chain (32, 33). Indeed, we observed a significantly higher proportion of IL-32^+^ cells among CD25^+^ activated compared to CD25^-^ non-activated cells in resting T cells from the blood of HDs (**Figures 4A, B**). In detail, IL-32 was expressed on average in 27% of CD3^+^CD25^+^, 23% of CD4^+^CD25^+^, and 48% of CD8^+^CD25^+^ T cells, in contrast to only 7% of CD3^+^CD25^-^, 7% of CD4^+^CD25^-^, and 8% of CD8^+^CD25^-^ T cells (**Figure 4B**). In agreement with this, after polyclonal stimulation 62% of CD3^+^CD25^+^, 60% of CD4^+^CD25^+^, and 61% of CD8^+^CD25^+^ T cells were IL-32-positive, whereas only 20%, 18%, and 21% of the respective CD25-negative compartment displayed IL-32 expression (**Figures 4A, C**). In conclusion, IL-32 expression is associated with an activated phenotype in T cells isolated from the blood of healthy individuals.

### T cells release IL-32β upon activation through TCR stimulation

**Figure 5:**
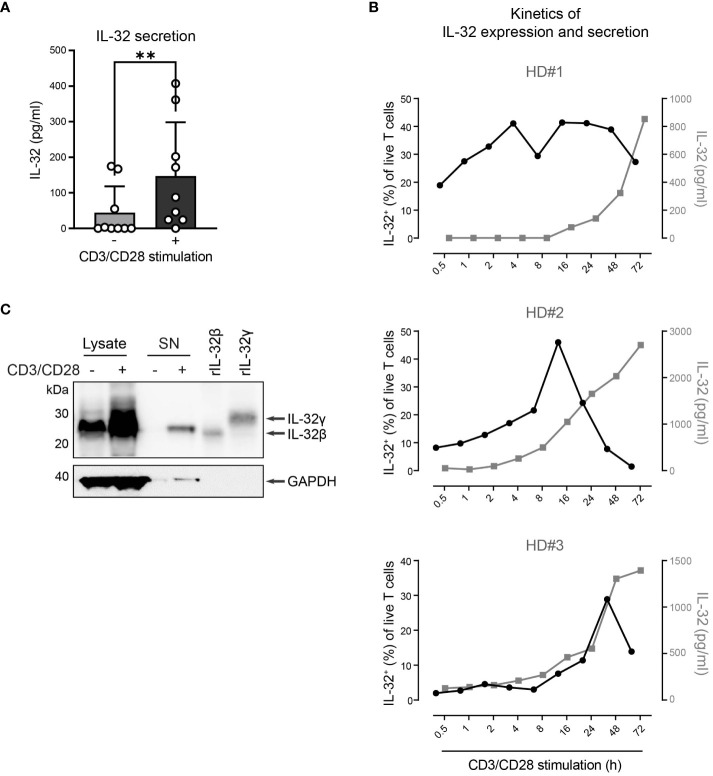
HD CD3^+^ T cells upregulate and secrete IL-32β after TCR stimulation. **(A)** IL-32 secretion by CD3^+^ T cells from healthy donors (HDs, n=9) after 24 h of culture without or with CD3/CD28 stimulation measured by ELISA analysis of culture supernatants. Mean+SD, dots depict data from individual experiments, Student’s paired t-test, **p<0.01. **(B)** Kinetics of the frequency of IL-32^+^ cells among all live T cells (%, black) in connection with the secretion of IL-32 (pg/ml, gray) in culture supernatants, as measured by ICFC and ELISA, respectively, over a time course of 0.5 – 72 h after CD3/CD28 stimulation of purified T cells from 3 different HDs. **(C)** WB analysis of IL-32 intracellular expression in total cell lysates (14 µg) and IL-32 secretion into the cell culture supernatant (SN; 14 µg, TCA/Ac enriched) by HD CD3^+^ T cells after 72 h of resting or CD3/CD28 stimulation next to human recombinant IL-32β (rIL-32β, 4 ng, MW: 23.1 kDa) and rIL-32γ (6 ng, MW: 28.1 kDa). As a loading control, the housekeeping gene GAPDH (MW: 37 kDa) was detected on the same blot. Representative data from n=2 HDs.

Next, we explored whether T cells do not only express IL-32β intracellularly but also release it into their surroundings. Therefore, we used IL-32 ELISA to analyze culture supernatants from T cells of healthy individuals after 24 h with or without polyclonal activation. Compared to resting conditions, TCR stimulation increased on average 3.3-fold the levels of IL-32 in T cell culture supernatants (**Figure 5A**, stimulated T cells: 147 pg/ml, resting T cells: 44 pg/ml). To understand the kinetics of IL-32 induction and release, we monitored the intracellular expression of IL-32 and its accumulation into the culture supernatants of T cells from three different HDs over a time course of 0.5 to 72 h after polyclonal stimulation. IL-32 expression kinetics varied among individual donors but the proportion of IL-32^+^ cells increased constantly, reaching maximum levels between 16 h and 48 h after stimulation (**Figure 5B**). Thereafter, the frequency of IL-32^+^ cells declined and coincided with the increased accumulation of IL-32 in culture supernatants. Thus, our data suggest that TCR stimulation induces the intracellular expression of IL-32 in T cells, which precedes its release into the culture supernatant.

To further validate this, we performed WB analysis of cell lysates and culture supernatants (SN) from T cells after resting or polyclonal stimulation for 72 h, when maximum IL-32 release was observed (**Figure 5B**). While both resting and stimulated T cells contained IL-32β in their cell lysates, though at different amounts, only TCR-stimulated T cells released IL-32 into the culture supernatant (**Figure 5C** and **Supplementary Figures 3A, B**). Taken together, our data demonstrate that resting T cells express constantly and at low levels the IL-32β protein isoform but require TCR stimulation for its intracellular accumulation and, subsequently, its release into the surrounding microenvironment.

### IL-2 induces IL-32 expression but TCR stimulation is required for its extracellular release

**Figure 6:**
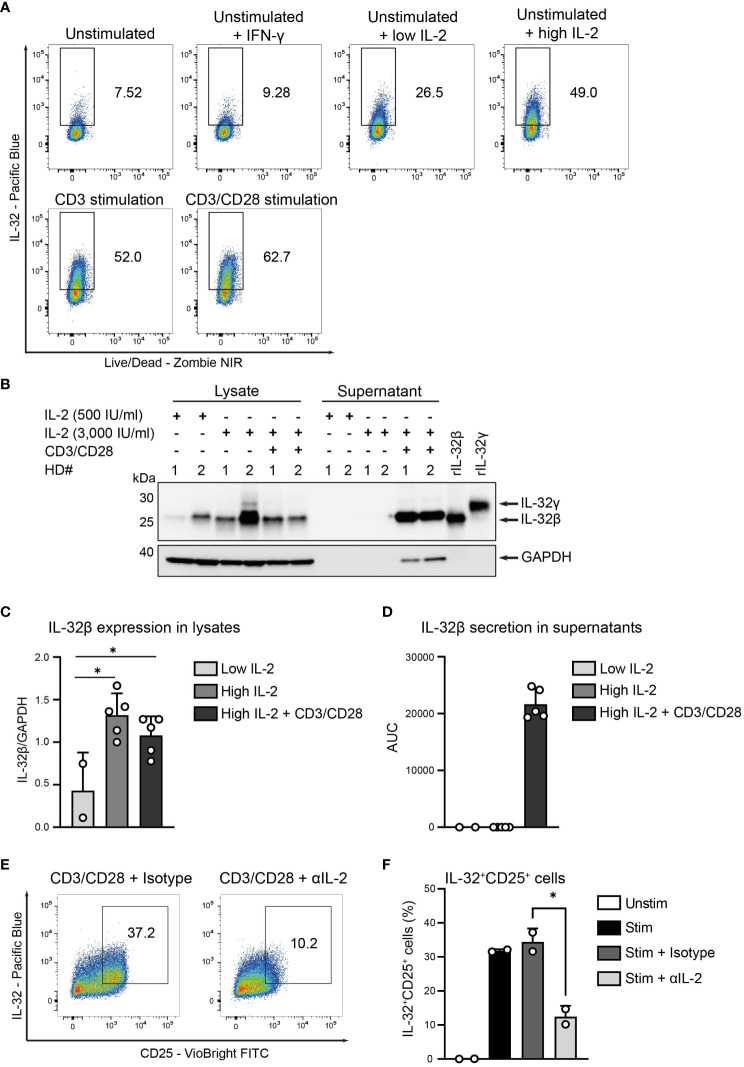
IL-2 induces IL-32 but TCR activation is required for IL-32 secretion. **(A)** IL-32 expression in CD3^+^ T cells from healthy donors (HDs) after 24 h of resting (unstimulated) either alone or in the presence of IFN-γ and low (100 IU/ml) or high (3,000 IU/ml) amounts of IL-2 and after 24 h of either CD3 or CD3/CD28 stimulation determined by ICFC analysis. Representative data from n=2 independent experiments. **(B–D)** WB analyses of IL-32 expression in cell lysates and IL-32 secretion into the cell culture supernatants (enriched via Amicon Ultracentrifugal filters) by HD CD3^+^ T cells after treatment for 48 h with 500 or 3,000 IU/ml IL-2 alone (low or high IL-2, respectively) or treatment with high IL-2 for 48 h with subsequent CD3/CD28 stimulation for 72 (h) **(B)** Representative blot of lysates and supernatants (15 µg) from n=2 HDs (#1, #2) next to rIL-32β (4 ng, MW: 23.1 kDa) and rIL-32γ (4 ng, MW: 28.1 kDa). As a loading control, the housekeeping gene GAPDH (MW: 37 kDa) was detected on the same blot. **(C)** Cumulative data of quantified IL-32β expression in cell lysates normalized to GAPDH for n=2–5 HDs, mean+SD, dots depict data from individual experiments, Student’s paired t-test, *p<0.05. **(D)** Cumulative data of quantified IL-32β secretion into culture supernatants displayed by the Area Under the Curve (AUC) for n=2–5 HDs, mean+SD. **(E, F)** IL-32 expression in HD T cells after resting (Unstim) or CD3/CD28 stimulation alone (Stim) or together with either an αIL-2 neutralizing antibody (Stim + αIL-2) or the respective isotype control (Stim + Isotype) for 24 (h) **(E)** Representative dot plots depicting IL32^+^CD25^+^ cells among live single T cells stimulated in the presence of the isotype or the αIL-2 antibody. **(F)** Cumulative data showing the frequency of IL-32^+^CD25^+^ cells among live single T cells, n=2 HDs, Student’s unpaired t-test, *p<0.05.

Based on the strong correlation of IL-32 and CD25 expression, we hypothesized that the IL-2 that is secreted by T cells during their stimulation (31) may also be involved in the induction of IL-32 and its release into the culture supernatant. Therefore, we cultured unstimulated T cells from HDs with either low (100 IU/ml) or high (3,000 IU/ml) amounts of IL-2 and compared their IL-32 expression to that obtained by TCR stimulation. We also treated unstimulated T cells with IFN-γ, since it is secreted together with IL-2 by Th1 Teff upon TCR stimulation (34) and induces IL-32 in human epithelial cells (1). While IFN-γ did not affect the expression levels of IL-32 on unstimulated T cells, IL-2 treatment increased IL-32 expression in a dose-dependent manner and to a similar degree as TCR stimulation (**Figure 6A**). Interestingly, IL-2 treatment alone, though at high dosage, did not trigger IL-32 release into the culture supernatant (**Figures 6B–D** and **Supplementary Figures 4A, B**). In contrast, TCR stimulation after high IL-2 treatment induced the enhanced accumulation of IL-32β within the culture supernatant (**Figures 6B, D** and **Supplementary Figures 4A, B**).

In addition, we stimulated HD T cells in the presence of anti-IL-2 neutralizing antibodies to investigate the role of IL-2 signaling in the induction of IL32-positive activated T cells. To ascertain efficient IL-2 neutralization, we assessed the levels of secreted IL-2 into culture supernatants of polyclonally stimulated T cells (820 pg/ml, **Supplementary Figure 5A**) and applied the neutralizing antibody at a concentration sufficient to block this amount of IL-2 (1X) or 10- and 100-fold higher amounts than this value (10X and 100X, respectively). Indeed, WB analyses of cell lysates demonstrated complete abrogation of phosphorylated STAT5 (pSTAT5) and, thus, IL-2-induced downstream signaling (35), already at the 1X concentration of the IL-2 neutralizing antibody compared to the respective isotype control (**Supplementary Figures 5B**, **6A, B**). Most importantly, IL-2 neutralization during T cell stimulation reduced significantly the generation of IL-32^+^CD25^+^ T cells 2.8-fold from 34.4% to 12.5% (**Figures 6E, F**). Taken together, our data demonstrate that T cells express IL-32 upon IL-2 signaling but require TCR stimulation for its extracellular release.

### T cells actively secrete IL-32β unconventionally through membrane pores and vesicular trafficking

Based on our data, IL-32β is the main protein isoform released into culture supernatants by stimulated T cells. However, it does not contain an N-terminal hydrophobic signal peptide, which allows secretion through the conventional secretory pathway, as in the case of IFN-γ (7, 36). While Goda *et al*. (18) suggested that T cells may release IL-32 only upon AICD, several studies have demonstrated that tumor cells can secrete IL-32 through extracellular vesicles (EVs) (2, 19). Therefore, we sought to understand the underlying mechanism of IL-32β release by T cells.

Most cytokines that contain a specific leader signal sequence are typically released through the conventional secretory pathways of constitutive secretion or regulated exocytosis of stored granules. During this process and before their release, proteins undergo processing in the endoplasmic reticulum (ER) and the Golgi apparatus (37). On the other side, leaderless proteins bypass the ER/Golgi network or may enter the ER but reach the plasma membrane in a manner independent of Golgi-derived COPI vesicles. However, the release of leaderless proteins may still be mediated through unconventional secretion pathways including both vesicular and non-vesicular mechanisms. Vesicular secretion can occur through exocytosis of endosomes or lysosomes, fusion of multivesicular bodies (MVB) or exosomes with the plasma cell membrane, microvesicle shedding/membrane blebbing or secretory autophagy. Non-vesicular models entail cytokine release through transporters located in the plasma cell membrane, membrane pore formation or passive diffusion upon cell lysis (37, 38).

**Figure 7:**
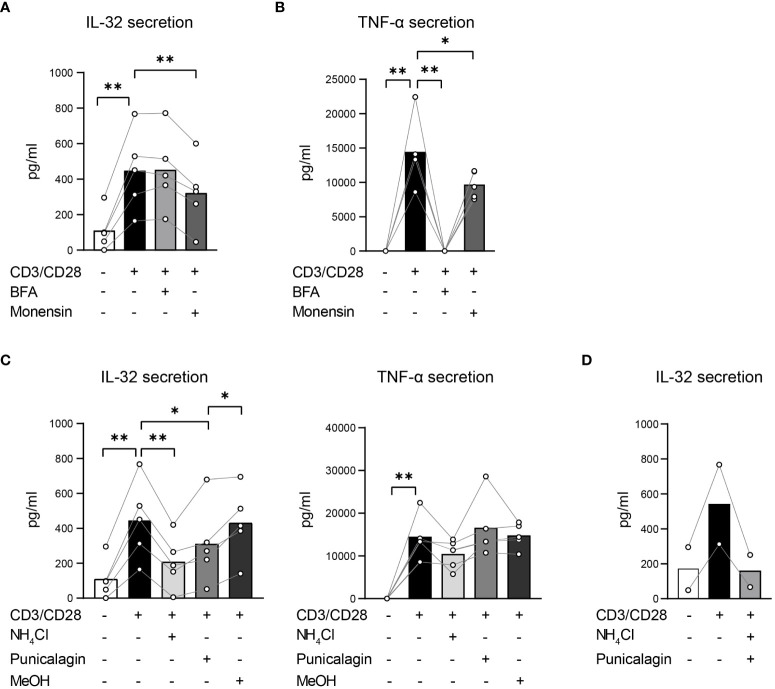
T cells actively secrete IL-32β mainly through unconventional secretion pathways bypassing the Golgi network. **(A, C, D)** IL-32 and **(B, C)** TNF-α secretion of Survivin T cells after 4 h of CD3/CD28 stimulation alone or in the presence of inhibitors that target **(A, B)** conventional or **(C, D)** unconventional protein secretion pathways analyzed by ELISA. **(A, B)** Treatment with Brefeldin A (BFA) or Monensin. **(C)** Application of ammonium chloride (NH_4_Cl), 2.5 µM Punicalagin or the respective volume of methanol (MeOH), the dissolution medium of Punicalagin, alone. **(D)** Combined treatment with NH_4_Cl and 2.5 µM Punicalagin. Related data within individual experiments are represented as dots, whereas each dot represents a mean of n=3 technical replicates. Individual experiments are connected by a line. Cumulative data of n=2–5 independent experiments, mean, Student’s paired t-test, *p<0.05, **p<0.01.

To identify which protein secretion pathways are utilized in T cells to secrete IL-32β, T cells were polyclonally stimulated either alone or in the presence of inhibitors of both conventional and unconventional pathways. To target the conventional protein secretion pathways, we applied the commonly used inhibitors Brefeldin A (BFA) and Monensin. BFA blocks the formation of COPI vesicles, disrupting the ER/Golgi transport of proteins (39), whereas Monensin interacts with the Na^+^/H^+^ transport, which is localized in the Golgi membrane, blocking the movement of proteins from medial- to trans-Golgi cisternae and, thus, their secretion (40). In addition, Monensin inhibits unconventional secretion pathways by neutralizing acidic intracellular compartments, including secretory lysosomes, pre-lysosomes and endosomes (41). To hinder specifically the unconventional protein secretion pathways, we supplemented the culture medium with ammonium chloride (NH_4_Cl) and Punicalagin. NH_4_Cl blocks protein trafficking into lysosomes by raising their intraluminal pH (42), resembling the effect of Monensin on the unconventional secretion pathway. Punicalagin, which is a complex polyphenolic compound isolated from pomegranate extracts, stabilizes membrane lipids, impairing lipid fluidity and cell membrane permeability (43). Consequently, it blocks the release of leaderless cytokines, such as IL-1β and IL-18, through cell membrane pores (44). To reduce interindividual variability in IL-32 expression and secretion, we used Survivin-specific T cells instead of T cells from HDs. Furthermore, Survivin T cells had been expanded in the presence of a high dose of IL-2 and show, therefore, elevated intracellular IL-32 expression, facilitating the interference with IL-32 secretion. Survivin T cells were stimulated using anti-CD3/CD28 antibodies in the presence of the inhibitors BFA, Monensin, NH_4_Cl or Punicalagin for 4 h and the cell-free culture supernatants were analyzed by IL-32 ELISA. IL-32 secretion by stimulated T cells was impaired significantly, although only partly, by Monensin, but remained unaffected by BFA (**Figure 7A**). To exclude that this was due to inefficient cytokine secretion blockade by the applied BFA concentration, we also analyzed the same culture supernatants by TNF-α ELISA, as TNF-α secretion is known to be inhibited by BFA (45). Indeed, BFA abrogated the secretion of TNF-α completely, while Monensin caused a partial but significant reduction (**Figure 7B**). Strikingly, NH_4_Cl decreased IL-32 secretion significantly to 46% of the levels produced by untreated stimulated T cells but had no impact on TNF-α secretion (**Figure 7C**). Previous studies have applied Punicalagin at 50 µM for efficient blockade of IL-1β secretion (44) and at an IC_50_ value of 3.91 µM to inhibit IL-1β secretion by Bone Marrow Derived Macrophages (BMDMs) (43). In analogy, we treated Survivin T cells with 50, 5 and 2.5 µM of Punicalagin. Since Punicalagin is dissolved in methanol (MeOH), which can lead to cell death, the respective amount of MeOH was also included as negative control for each applied concentration. As Punicalagin inhibits membrane permeability, in high concentrations and after long exposure it may also completely hinder the overall cytokine secretion mechanisms in T cells. To exclude this possibility, we analyzed culture supernatants not only for IL-32 but also for TNF-α. While TNF-α secretion was completely hindered at high concentrations of Punicalagin (50 µM), TNF-α levels remained unaffected with 2.5 and 5 µM of Punicalagin (**Figure 7C** and **Supplementary Figure 7**). In contrast, IL-32 secretion was significantly diminished with 2.5 and 5 µM Punicalagin to 69% and approximately half (53%), respectively, of the levels secreted by untreated stimulated Survivin T cells (**Figure 7C** and **Supplementary Figure 7**). Moreover, the combined treatment with Punicalagin and NH_4_Cl completely abrogated IL-32 secretion of stimulated T cells to the baseline level of resting T cells (**Figure 7D**). Taken together, our data demonstrate that T cells actively secrete IL-32β predominantly in an unconventional manner, most probably through membrane pores. Moreover, they show that IL-32 secretion involves its intracellular processing along vesicular trafficking routes, raising the possibility that IL-32 may also be released through extracellular vesicles.

### IL-32β is secreted by T cells predominantly as a free protein

**Figure 8:**
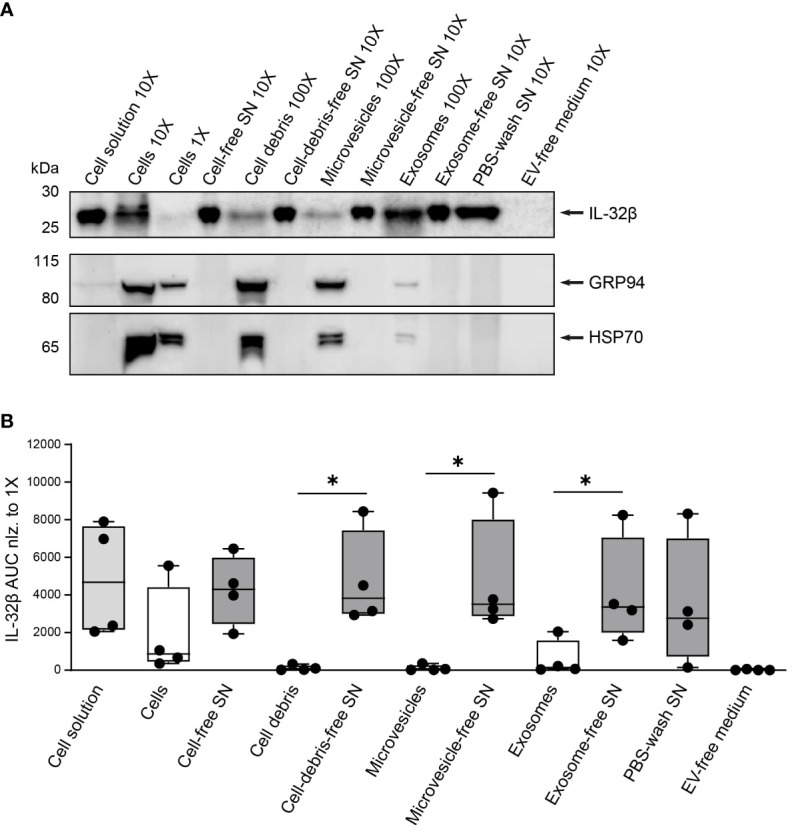
IL-32 is secreted by T cells predominantly as a free protein. **(A)** Representative WB analyses of IL-32β and the microvesicle/exosome markers GRP94 and HSP70 in lysates of cells, cell-debris, microvesicles and exosomes, into the respective cell solution and cell-free, cell debris-free, microvesicle-free and exosome-free supernatant (SN), the PBS-wash SN, which was used to wash the exosome pellet, and in the EV-free medium used to culture Survivin-specific T cells for 4 h with anti-CD3/CD28 antibodies. The loaded quantity of each lysate and SN sample corresponds to 0.16x10^6^ (1X), 1.6x10^6^ (10X) and 16x10^6^ (100X) Survivin-specific T cells and the amount of PBS-wash SN derived from this cell number or the volume of EV-free medium used to culture this cell number. MW in kDa (IL-32β: 23.1, GRP94: 98, HSP70: 70). IL-32β, GRP94, HSP70: n=4. n represents independent experiments. **(B)** Cumulative data of quantified IL-32β expression in lysates and supernatants displayed by the Area Under the Curve (AUC) for n=4 independent experiments normalized to 0.16x10^6^ Survivin-specific T cells. Dots depict data from individual experiments, Student’s paired t-test, *p ≤ 0.05.

To determine to what degree T cells release IL-32β as a free protein and whether and to what extent in the form of vesicles, we assessed the presence of IL-32β in EVs, particularly microvesicles and exosomes, derived from stimulated T cells in comparison to the respective vesicle-free supernatants. Therefore, Survivin T cells were stimulated with anti-CD3/CD28 antibodies for 4 h and the resulting cell solution was first depleted from cells, generating cell-free supernatant (SN), and, in a second step, from cell-debris using centrifugation. The resulting cell-debris-free SN was further centrifuged to remove microvesicles, producing microvesicle-free SN. The latter was, in turn, separated by ultracentrifugation into exosomes and exosome-free SN. Importantly, the same ultracentrifugation process was also applied on the culture medium that was used for T cell stimulation cultures, to exclude any unspecific background resulting from contaminating vesicles in media serum (EV-free medium). Isolated cells, cell-debris, microvesicles and exosomes were first washed with PBS and (ultra)centrifuged to exclude any contaminating supernatant-derived proteins. The lysates of the obtained pellets were analyzed by WB against IL-32 next to the complete cell solution but also cell-, cell debris-, microvesicle- and exosome-free supernatants, while EV-free medium was also included as a negative control. To ascertain that the process of microvesicle and exosome elimination from T cell culture supernatants was efficient, we analyzed the same lysates and supernatants by WB for common vesicular/exosomal markers, including HSP90B1 (also known as GRP94) and HSP70 (46). Both markers were detected in lysates from cells, cell debris, microvesicles and exosomes. However, neither of the two markers was identified in the exosome-free medium nor in the EV-free medium, which was used to culture T cells, or the supernatant derived after washing exosomes with PBS and ultracentrifugation (PBS-wash SN) (**Figure 8A** and **Supplementary Figures 8A, B**). Thus, the followed approach of serial centrifugation and ultracentrifugation steps completely removed microvesicles and exosomes from the tested supernatants. This procedure revealed that exosomes contain increased amounts of IL-32 compared to microvesicles and cell debris derived from the same number of stimulated T cells (**Figures 8A, B** and **Supplementary Figures 8A, B**). However, the highest amount of IL-32 (92%) was detected in exosome-free SNs at levels ~30-fold higher than the ones detected in exosomes isolated from the same T cells and to a similar degree in the PBS-wash SN of exosomes (**Figure 8B**). Taken together, our data demonstrate that IL-32β is actively secreted by T cells upon TCR stimulation, in a minor part through exosomes, but predominantly as a free protein.

## Discussion

Here we show that human T cells express the IL-32β isoform upon IL-2 receptor signaling, while TCR stimulation triggers its active secretion as a free protein through the plasma membrane in an unconventional manner, most probably through membrane pores. Besides, T cells release a smaller amount of IL-32β in EVs. Since these can effectively mediate intercellular protein exchange (47), IL-32β-secreting T cells may directly affect inflammatory responses of cells in their microenvironment. Activated T cells constitute a major cell compartment in the blood and in the lymphatic organs (48) and may, therefore, represent a considerable source of systemic IL-32.

Indeed, increased IL-32 levels correlate with infectious diseases (3, 4), acute and chronic inflammation (1) and inflammation-related diseases, like skin inflammation (49), allergy (50) and atherosclerosis (51), but also cancer development (7) and disease progression in several autoimmune disorders, like Rheumatoid Arthritis (RA), Inflammatory Bowel Disease and Type 1 Diabetes (15). In these diseases pro- and anti-inflammatory functions of IL-32 have been described (1–4, 15, 17, 49–51) and IL-32 has, thus, emerged as a biomarker and potential therapeutic target for blocking antibodies, small molecule inhibitors and deubiquitinase inhibitors that inhibit ubiquitin removal from IL-32, inducing its proteasomal degradation (52). Furthermore, targeted reduction of IL-32 expression and, consequently, its secretion by T cells could favor patients suffering from diseases, in which T cells are the major IL-32 producers, like HIV-infection, beta cell autoimmunity and cancer (8–10).

*IL32β* was the most abundant isoform transcribed in CD3^+^ T cells from healthy individuals but also in intratumoral CD4^+^ Th subsets and circulating TA-specific CD4^+^ Teff from breast cancer patients, CD4^+^ and CD8^+^ MILs from a multiple myeloma patient and TA Survivin-specific CD8^+^ Teff cells. To a much lesser extent, these T cells also expressed *IL32C(η)* and the starting sequence *IL32γ*. We did not observe major differences in the relative distribution of *IL32* isoforms among the tested T cell subsets, but they displayed substantial differences in the overall amount of *IL32β* expression. Immune suppressive CD4^+^ Treg and Th1 Teff in breast tumors showed the highest expression of *IL32β*, followed by TA-specific CD4^+^Teff in the blood of breast cancer patients. Since IL-32β represents an isoform with a proposed dominant immune suppressive capacity (15), the extent of its expression and release may contribute to the capacity of T cells to regulate inflammatory signaling in the course of an immune response.

Hitherto, no isoform-specific antibodies against IL-32 are available, limiting analyses of different IL-32 isoforms on the protein level and our understanding of their functions. As a result, previous studies have addressed IL-32 expression in general without defining specific IL-32 isoforms. In a previous attempt to address this issue, Goda *et al.* (18) compared lysates from human T cells with lysates from *IL32β or IL32γ*-transfected HEK293T cells in western blots stained with anti-IL-32 serum. Despite difficult interpretation of the data due to multiple detected bands and almost identical IL-32 WB profiles between *IL32γ*- and *IL32β*-transfected HEK293T cells, they concluded in favor of an IL-32β production by T cells (18). We here clarified this assumption by WB analysis of T cell lysates and supernatants using an anti-IL32αβγδ antibody next to the pure recombinant form of each isoform (rIL-32β, rIL-32γ) separately, allowing a side-by-side comparison of their molecular weights. Our analysis revealed that T-cell derived IL-32 bands have the same molecular weight as rIL-32β but not as rIL-32γ, thereby proving that IL-32β is the predominant protein isoform expressed in CD3^+^ T cells and TA Survivin-specific CD8^+^ T cells from healthy individuals.

In epithelial cells IL-32 expression is triggered through the IFN-γ- or the TNF-receptor (2). We here demonstrate that in T cells, IL-32 expression is largely triggered by stimulation of the IL2R. This is evident from the following observations. IL-32 expression was predominantly confined to activated T cells expressing the high affinity IL-2 receptor alpha chain CD25 (32, 33), irrespective of a preceding TCR stimulation. IL-2 exposure alone triggered IL-32 expression in a dose-dependent manner to levels comparable with TCR stimulation. On the other hand, IFN-γ alone had only a minor impact on IL-32 expression by T cells despite its known capacity to induce IL-32 in human epithelial cells (1). Moreover, IL-32 expression of TCR stimulated T cells was enhanced upon costimulation through CD28, which triggers increased secretion of IL-2 compared to IFN-γ or TNF-α (31). Finally, IL-2 neutralization during TCR stimulation reduced significantly by ~73% the proportion of T cells that co-expressed IL-32 and the high-affinity IL-2 receptor (32). This demonstrates that IL-32 induction in T cells depends largely on IL-2 receptor ligation. Thus, IL-2 is a major inducer of IL-32 expression by T cells. The induction of IL-32 expression by TCR stimulation alone can, therefore, be explained by the fact that TCR stimulation triggers not only IL-2 secretion (31) but also the expression of its high-affinity receptor CD25 (32, 33). However, IL-2 neutralization did not completely inhibit IL-32 expression, despite effective abrogation of the IL-2 dependent STAT5 phosphorylation, and we further detected intracellular IL-32 expression in a small fraction of CD25^-^ T cells. Therefore, we cannot exclude that IL-32 upregulation can be also mediated through other receptors that recognize TCR-induced ligands not tested in this study, such as TNF-α (31). For example, Pan *et al.* identified NF-κB binding sites within the promoter region of the *IL32* gene (53), suggesting that TCR mediated NF-kB signaling (54) may also drive IL-32 expression to some extent. The IL-2 dependence of IL-32 induction is in accordance with our and other observations that IL-32 expression is highest in Treg (14, 15), since these are CD25^+^ by definition (12).

The functional role of IL-32β with its ambiguous effects in T cell responses remains largely elusive. However, repetitive TCR stimulation commonly leads to upregulation of co-inhibitory molecules in T cells, such as PD-1 or CTLA-4, as a negative regulation mechanism to control overwhelming immune responses (55). Taking this into consideration, TCR-induced IL-32 may participate in regulating T cell homeostasis, expansion, and function in the context of an immune response.

Besides a T-cell intrinsic regulatory role, IL-32β may also exert important cell-extrinsic regulatory functions within the tissue microenvironment or even systemically. We found that IL-32β is the predominant protein isoform in T cell culture supernatants. This is in contrast to the common notion that only the IL-32γ isoform may be secreted, as it solely contains a hydrophobic signal sequence cleavage site for protein secretion (7). Nevertheless, IL-32β contains a tyrosine sulfation site (1), a post-translational modification characteristic of secreted and transmembrane-spanning proteins (56). Indeed, IL-32 has been detected both in human serum and culture supernatants of various cell types, supporting its release (2). Several mechanisms have been suggested for extracellular IL-32 accumulation, including secretion of IL-32γ by RA synovial fibroblasts (57) and the release of IL-32 by intestinal epithelial cells via multi-vesicular bodies and exosomes (2). In our attempt to clarify this question, we studied a potential release via extracellular vesicles using serial (ultra)centrifugation of T cell culture supernatants and different inhibitors targeting both conventional and unconventional protein secretion pathways in stimulated T cells. These analyses revealed that the vast amount, namely approximately 92%, of the extracellular IL-32β is not released with extracellular vesicles but directly through an unconventional secretory pathway. Exosomes contained most of the remaining IL-32β fraction, while only traces were found in microvesicles and cell debris. IL-32β secretion did not involve the Golgi apparatus since it was not inhibited by BFA (39). In contrast, IL-32β secretion from activated T cells was significantly impaired, almost to half of the original value of untreated T cells, by low concentrations of Punicalagin, which reduces cell membrane fluidity and blocks the formation of cell membrane pores, inhibiting unconventional protein secretion of leaderless cytokines, like IL-1β and IL-18 (43, 44). High concentrations of Punicalagin result in a universal tightness of the plasma membrane, as demonstrated by the complete blockade of not only IL-32β but also TNF-α secretion. However, IL-32β secretion was still significantly hindered at low Punicalagin concentrations, which did not affect TNF-α secretion, supporting the interpretation that the cell membrane fluidity is a crucial prerequisite for direct IL-32β secretion by T cells, most probably through membrane pores.

In addition, IL-32β levels were partly decreased by Monensin, a common inhibitor of multiple intracellular Na^+^/H^+^ channels (40), demonstrating the active secretion of IL-32β by activated T cells. However, such channels are also expressed in acidic intracellular compartments, including secretory lysosomes but also pre-lysosomes and endosomes, and are essential for maintaining their low pH (41). Thus, an impact of intracellular vesicular protein trafficking on IL-32 recirculation and release cannot be excluded. In accordance, ammonium chloride, which alkalizes intracellular vesicles (42), inhibited IL-32β release to more than half of its original value and to a greater extent than Monensin. Although IL-32β is leaderless, its translocation into vesicles may occur through the protein channel TMED10, which is located at the (ER)-Golgi intermediate compartment and facilitates the vesicle entry of several cargoes, including the cytokine IL-1β (58). Indeed, several studies have demonstrated the presence of IL-32 in intracellular membrane vesicles of cancer cells and postulated the secretion of IL-32 through EVs (2, 19). Such tumor-cell derived EVs can be taken up by macrophages, resulting in their polarization toward the immune-suppressive M2 type (19). Thus, our findings indicate that IL-32β is subject to the intracellular protein trafficking and recirculation system within vesicles, which may affect its processing and stability, but that in contrast to other cell types such as epithelial cells (2, 19), T cells seem to secrete the vast majority of IL-32 not through EVs but directly through the plasma membrane.

The concluding concept of our study – the mainly active direct secretion of IL-32β by T cells - is in conflict with a previous report of T-cell-mediated IL-32 release due to AICD (18). This was mainly based on the observed correlation between elevated IL-32 and increased levels of the cytosolic protein GAPDH in culture supernatants of stimulated T cells. In agreement with this, we also detected GAPDH proteins in IL-32β-containing culture supernatants of activated T cells. However, GAPDH can also be secreted independent of cell lysis and death (59). Macrophages, in particular, release GAPDH through an unconventional pathway after activation of the ATP-gated cation channel P2X7 receptor (P2X7R) by extracellular ATP (60). P2X7R stimulation triggers various secretion pathways, including plasma membrane blebbing, exocytosis of lysosomes/exosomes and the generation of membrane pores large enough to allow the flux of macromolecules (61). Interestingly, P2X7R initiates the non-classical secretion of the leaderless cytokines IL-1β and IL-18 (62), which is inhibited by Punicalagin (43, 44) that also impaired IL-32 secretion by activated T cells in our study. Early upon infection and acute tissue trauma P2X7R is upregulated on immune effector cells, including T cells, while ATP is released through trauma-induced cell lysis and several other non-lytic mechanisms (62). Thus, TCR-stimulated T cells most probably secrete GAPDH along with IL-32β in an unconventional manner.

In conclusion, our study demonstrates that T cells predominantly produce the IL-32β protein upon ligation of the IL-2 receptor and actively secrete this isoform after TCR stimulation in an unconventional manner, including vesicular trafficking, but predominantly as a free protein through a pathway that depends on plasma membrane fluidity. Further studies investigating in depth the secretion route of IL-32β in T cells, its uptake mechanisms by recipient cells and its impact on their biological processes and functions will help to understand the role of T-cell derived IL-32β in inflammation and immune responses during infection, allergy, autoimmunity, and cancer.

## Data Availability

Publicly available datasets were analyzed in this study. The single-cell transcriptome datasets analyzed for this study can be found in the European Genome-Phenome Archive under the accession code EGAD00001004069 (https://www.ebi.ac.uk/ega/datasets/EGAD00001004069).
