# Supplementary material for: IL-2 and TCR stimulation induce expression and secretion of IL-32β by human T cells
Source: Front Immunol. 2024 Aug 15;15:1437224. doi: 10.3389/fimmu.2024.1437224 (PMC11357969; doi:10.3389/fimmu.2024.1437224)
Supplement: Supplementary file 1 [file DataSheet1.pdf]

Supplementary Material

Supplementary Tables

Supplementary Table 1. PCR primers for *IL32*-isoform expression using RT-qPCR

Gene	Position	Sequence (5' → 3')	T_M (°C)	T_A (°C)	Amplicon length (bp)
ACTB	forward	AGAAAATCTGGCACCACA	69.0	59	125
	reverse	GGGGTGTTGAAGGTCTCAAA	66.7		
IL32	forward	GGAGACAGTGGCGGCTTAT	64.2	59	83
	reverse	GGCACCGTAATCCATCTCTT	62.8		
IL32α (A)	forward	CACCCAGAGCTCACTCCTCT	63.7	59	138
	reverse	GGCTCCGTAGGACTTGTCAC	63.7		
IL32β (B)	forward	GAAGACTGCGTGCAGAAGGT	64.6	59	149 & 172
	reverse	CTTTCTATGGCCTGGTGCAT	63.9		
IL32γ (E)	forward	TACTTCTGCTCAGGGGTTGG	64.1	59	271
	reverse	TGGGTGCTGCTCCTCATAAT	64.5		
IL32D	forward	AGGACGTGGACAGGACGACT	66.2	59	85
	reverse	AGGAGTGAGCTCTGGGTGCT	65.5		
IL32η (C)	forward	AGGCCCGAATGGTGATGT	65.5	59	144
	reverse	GGCACCGTAATCCATCTCTT	62.8		
PPIB	forward	GATGGCACAGGAGGAAAGAG	63.8	59	71
	reverse	AGCCAGGCTGTCTTGACTGT	64.1		

Supplementary Table 2. PCR program for *IL32*-isoform expression using RT-qPCR

Step	Temperature (°C)	Time (hh:mm:ss)	Cycle #	Ramp rate (°C/s)
Denaturation	95	00:05:00	1	2.74
PCR stage	95	00:00:10	40	2.74
	59	00:00:30		2.12
	72	00:00:10		2.12
Melt curve stage	95	00:00:15	1	2.74
	59	00:01:00		2.12
	95	00:00:15		0.15
	60	00:00:15		1.6
Final hold	4	00:60:00	1	1.6

Supplementary Table 3. Antibodies for Flow Cytometry and Western Blot analysis

Target Molecule	Trade name	Catalog Number	Company	RRID
CD25	anti-CD25-VioBright-FITC	130-113-283	Miltenyi Biotec	AB_2734062
CD28	anti-CD28 antibody	302902	BioLegend	AB_314304
CD3	anti-CD3 antibody	14-0037-82	eBioscience	AB_467057
CD3	anti-CD3-BV605	300459	BioLegend	AB_2564379
CD4	anti-CD4-PerCP	300528	BioLegend	AB_893321
GAPDH	anti-GAPDH	sc-47724	Santa Cruz Biotechnology	AB_627678
GRP94	anti-GRP94	ADI-SPA-850-D	Enzo Life ScienceS	AB_2039133
HSP70	anti-HSP70	610607	BD Biosciences	AB_397941
IgG	HPR-coupled anti-mouse IgG	7076S	Cell Signaling	NA
IgG	HPR-coupled anti-rat IgG	7077	Sigma	NA
IgG	HPR-coupled anti-rabbit IgG	7074S	Cell Signaling	NA
IgG1	Mouse IgG1 Isotype Control	MAB002	R&D Systems	AB_357344
IgG1,κ	mIgG1,κ-Pacific Blue™ isotype control	400131	BioLegend	AB_2923473
IgG2b,κ	mIgG2b,κ-VioBright-FITC isotype control	130-104-649	Miltenyi Biotec	AB_2661748
IL-2	Human anti-IL-2 antibodies	MAB202R-SP	R&D Systems	NA
IL-32αβγδ	anti-IL-32αβγδ-Pacific Blue™	513501	BioLegend	AB_2124018
IL-32αβγδ	Purified anti-human IL-32αβγδ	513501	BioLegend	AB_2124018
pSTAT5 (Tyr694)	anti-pSTAT5 (Tyr694)	9359S	Cell Signaling	NA

NA: Not available

Supplementary Table 4. Commercial Kits

Kit name	Catalog Number	Company
BD Pharmingen™ Transcription Factor Buffer Set	562574	BD Biosciences
cOmplete™, EDTA-free Protease Inhibitor Cocktail	04693132001	Roche, Merck Millipore
Dynabeads™ Untouched™ Human T Cells Kit	11344D	Thermo Fisher Scientific
eBioscience™ Foxp3/Transcription Factor Fixation/Permeabilization Concentrate and Diluent	00-5521-00	eBioscience
Human IFN-γ ELISA Set	555142	BD Biosciences
Human IL-32 DuoSet ELISA	DY3040-05	R&D Systems
MILLIPLEX Human CD8+ T Cell MAGNETIC Premixed 17 Plex Kit	HCD8MAG15K17PMX	Merck
P3 Primary Cell 4D Nucleofector X Kit S	V4XP-3032	Lonza
Phosphatase Inhibitor Cocktail 3	P0044-1ML	Merck Millipore
Pierce™ BCA Protein Assay Kit	23225	Thermo Fisher Scientific
Pierce™ ECL Western Blotting Substrate	32209	Thermo Fisher Scientific
Protease Inhibitor Cocktail Set III, EDTA-Free	539134-1ML	Merck Millipore
QuantiFast® SYBR® Green PCR Kit	204057	Qiagen
QuantiNova® SYBR® Green PCR Kit	208056	Qiagen
QuantiTect® Reverse Transcription Kit	205313	Qiagen
RNeasy® Micro Kit	74004	Qiagen
RNeasy® Mini Kit	74106	Qiagen
Trans-Blot® Turbo™ RTA Mini 0.45 µm Nitrocellulose Transfer Kit	1620115	Bio-Rad
Trident femto Western HRP Substrate	GTX14698	GeneTex
Zombie Aqua™ Fixable Viability Kit	423102	BioLegend
Zombie NIR™ Fixable Viability Kit	423105	BioLegend

Supplementary Figures

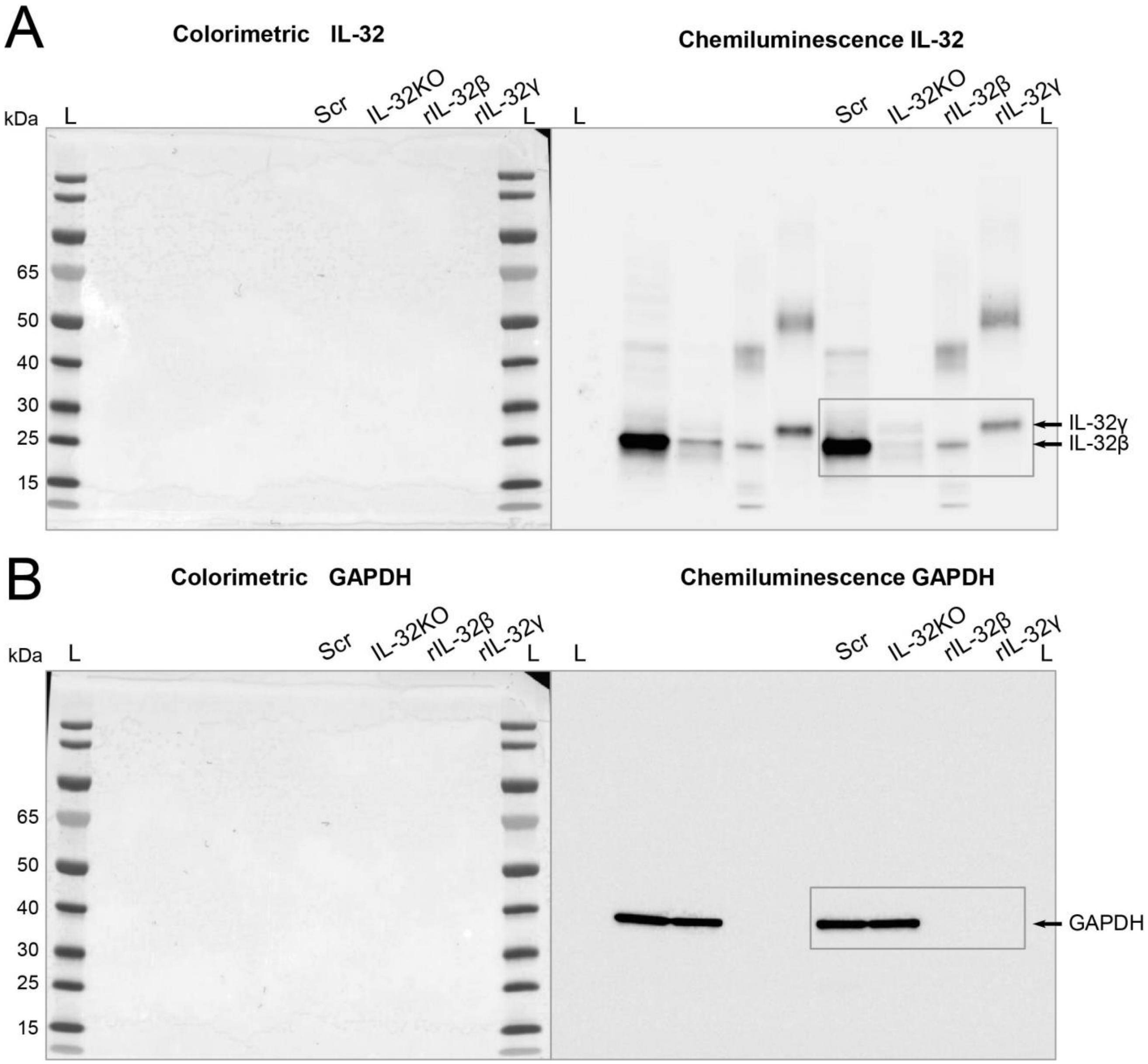

Supplementary Figure 1. Original Western Blot membranes corresponding to main Figure 1E. WB analysis of **(A)** IL-32 and **(B)** GAPDH expression in cell lysates (27 µg) of Scr and IL-32KO Survivin T cells next to rIL-32β (4 ng, MW: 23.1 kDa) and rIL-32γ (4 ng, MW: 28.1 kDa). Colorimetric images depict the used protein ladder (L), while Chemiluminescence images present the proteins of interest. Grey squares highlight the blot area presented in Figure 1E. MW: Molecular Weight.

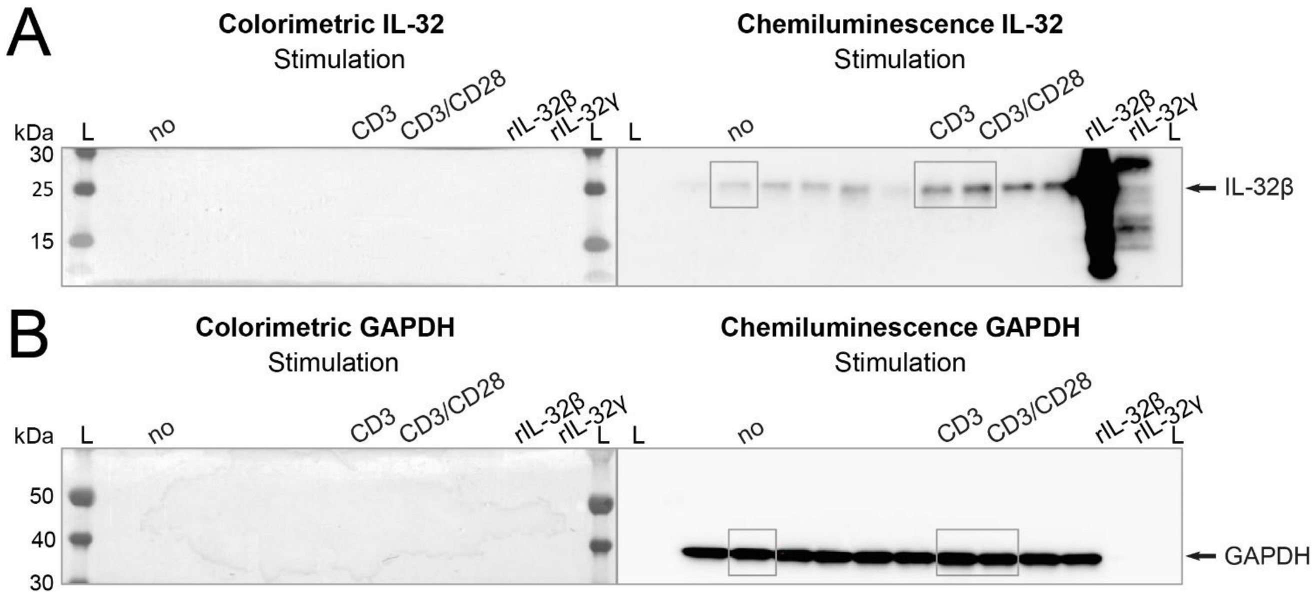

Supplementary Figure 2. Original Western Blot membranes corresponding to main Figure 2C. WB analysis of **(A)** IL-32 and **(B)** GAPDH expression in cell lysates from unstimulated HD T cells compared to stimulation with anti-CD3 or anti-CD3/CD28 antibodies next to rIL-32β (MW: 23.1 kDa) and rIL-32γ (MW: 28.1 kDa). Colorimetric images depict the used protein ladder (L), while Chemiluminescence images present the proteins of interest. Grey squares highlight the blot area presented in Figure 2C. MW: Molecular Weight.

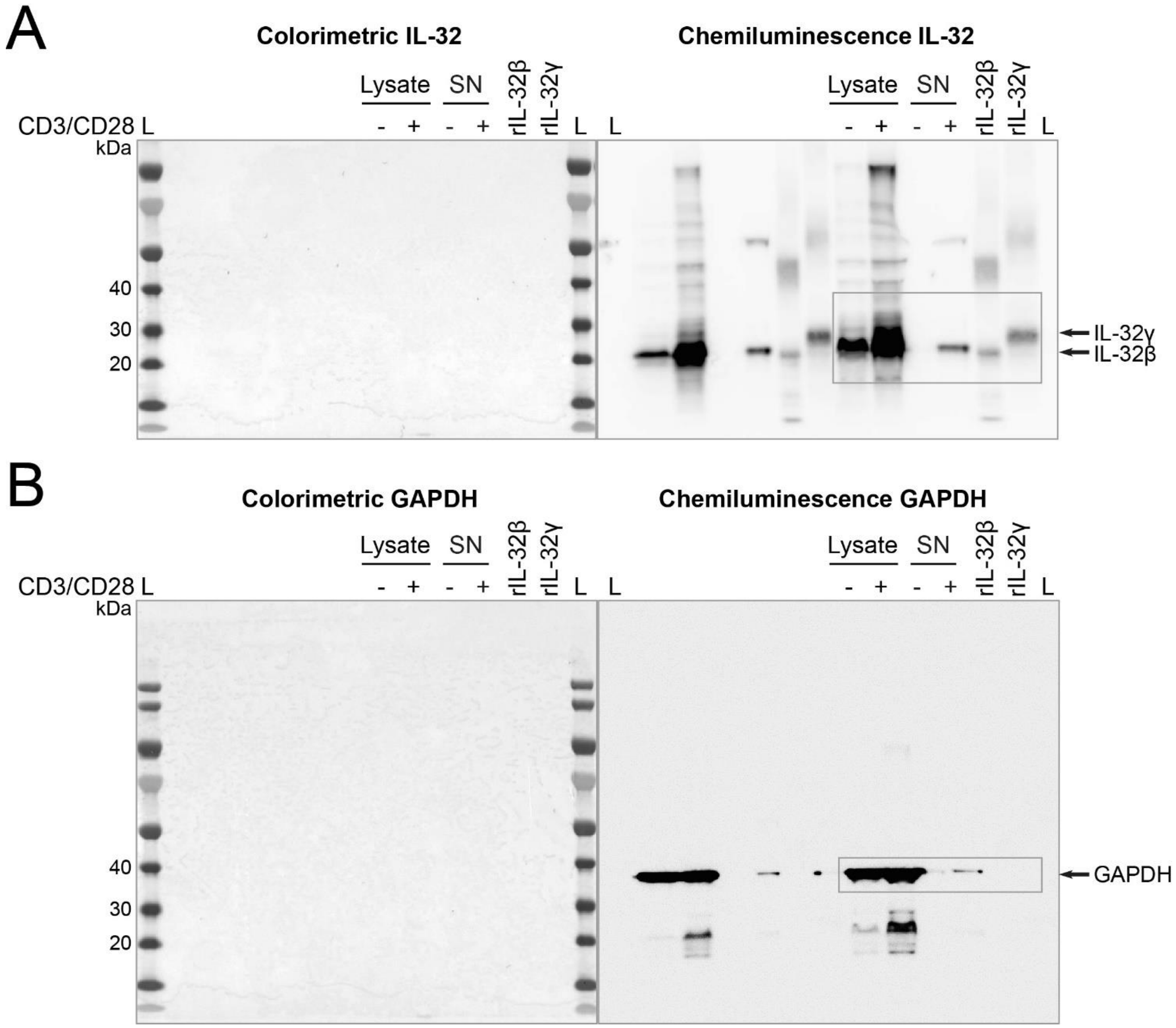

Supplementary Figure 3. Original Western Blot membranes corresponding to main Figure 5C. WB analysis of **(A)** IL-32 and **(B)** GAPDH expression in total cell lysates (14 µg) and secretion into the cell culture supernatant (SN; 14 µg, TCA/Ac enriched) by HD $CD3^+$ T cells after 72 h of resting or CD3/CD28 stimulation next to human recombinant IL-32β (rIL-32β, 4 ng, MW: 23.1 kDa) and rIL-32γ (6 ng, MW: 28.1 kDa). Colorimetric images depict the used protein ladder (L), while Chemiluminescence images present the proteins of interest. Grey squares highlight the blot area presented in Figure 5C. MW: Molecular Weight.

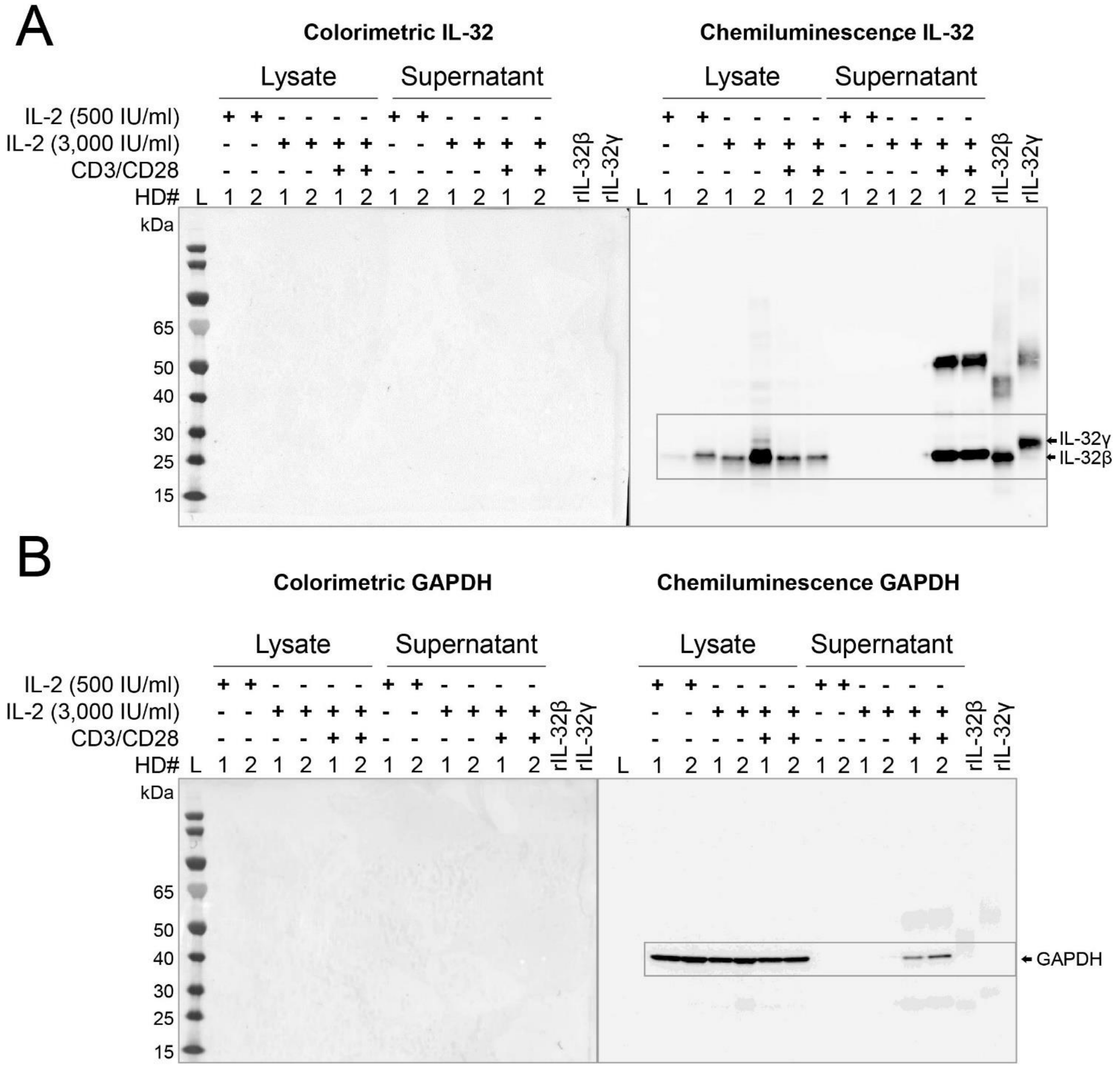

Supplementary Figure 4. Original Western Blot membranes corresponding to main Figure 6B. WB analysis of **(A)** IL-32 and **(B)** GAPDH expression in cell lysates and secretion into the cell culture supernatants (enriched via Amicon Ultracentrifugal filters) by HD CD3^{+} T cells after treatment for 48 h with 500 or 3,000 IU/ml IL-2 alone (low or high IL-2, respectively) or treatment with high IL-2 for 48 h with subsequent CD3/CD28 stimulation for 72 h. **(A,B)** Representative blot of lysates and supernatants (15 µg) from n=2 HDs (#1, #2) next to rIL-32β (4 ng, MW: 23.1 kDa) and rIL-32γ (4 ng, MW: 28.1 kDa). The housekeeping gene GAPDH (MW: 37 kDa) served as a loading control. Colorimetric images depict the used protein ladder (L), while Chemiluminescence images present the proteins of interest. Grey squares highlight the blot area presented in Figure 6B. MW: Molecular Weight.

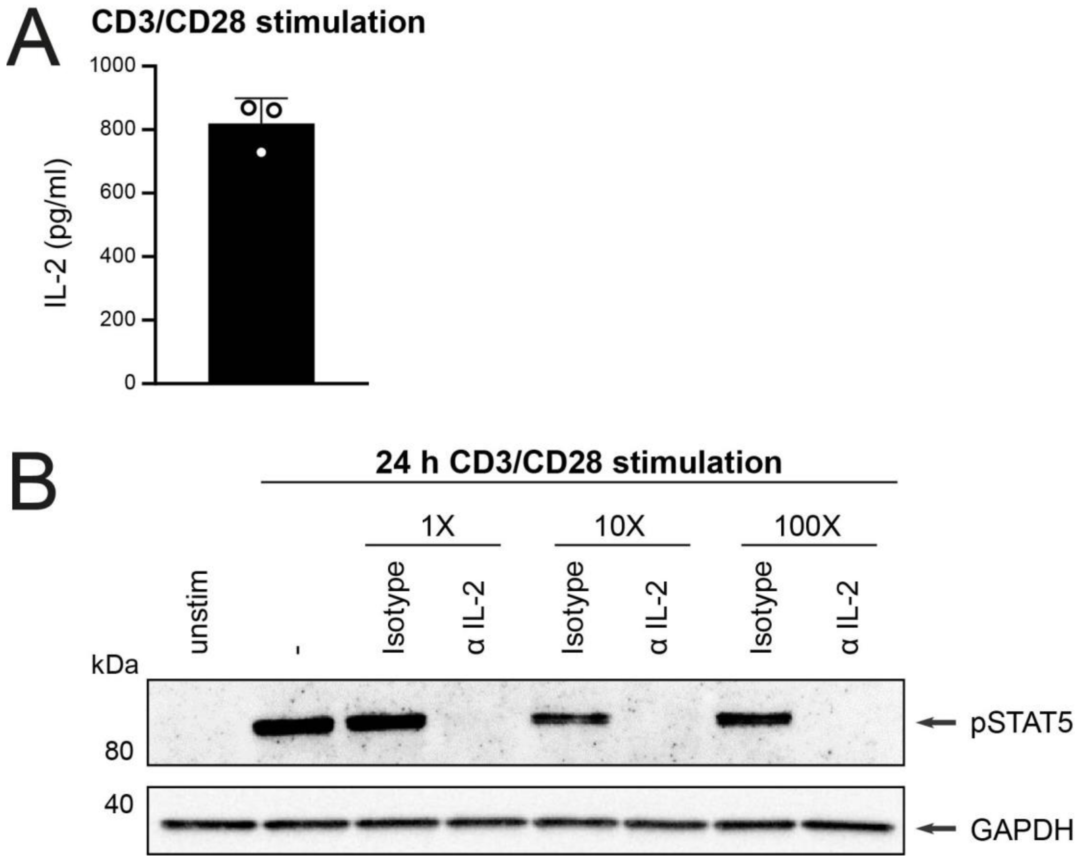

Supplementary Figure 5. Inhibition of STAT5 signaling using αIL-2 neutralizing antibody. (**A**) IL-2 concentration in HD T cell culture supernatants after CD3/CD28 stimulation determined using the Luminex multiplex cytokine assay MILLIPLEX Human CD8^{+} T Cell MAGNETIC Premixed 17 Plex Kit. Cumulative data from n=3 independent experiments, Mean+SD, dots depict data from individual experiments. (**B**) Representative WB analysis of phosphorylated STAT5 (MW: 90 kDa) and the housekeeping gene GAPDH (MW: 37 kDa), as a loading control, in CD3^{+} T cells from one healthy donor (HD) after the application of an αIL-2 neutralizing antibody or the respective isotype control during CD3/CD28 stimulation for 24 h. 1X, 10X, 100X represent the titrated concentration of neutralizing antibodies according to the expected amount of secreted IL-2. n=2 independent experiments. MW: Molecular weight.

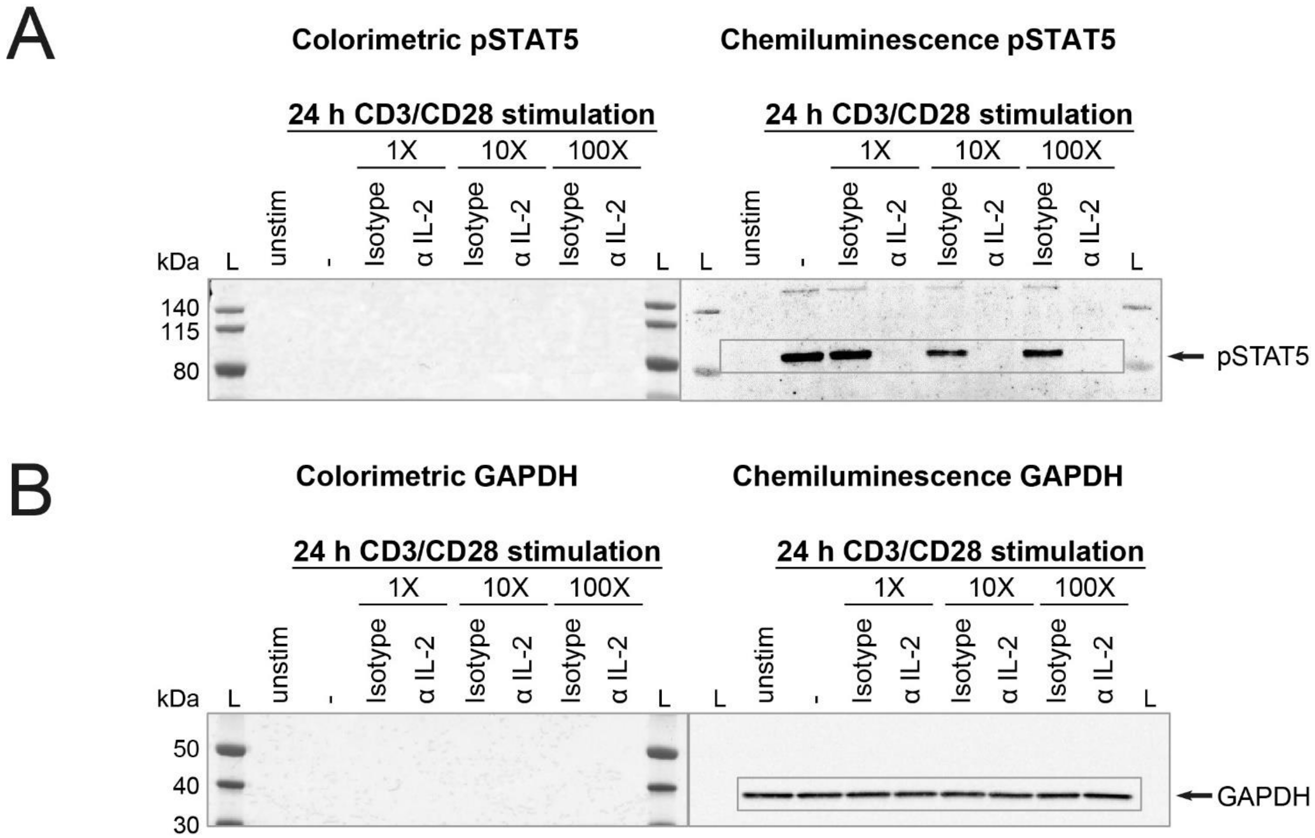

Supplementary Figure 6. Original Western Blot membranes corresponding to Supplementary Figure 5B. WB analysis of **(A)** phosphorylated STAT5 and **(B)** the housekeeping gene GAPDH, as a loading control, in $CD3^+$ T cells from one healthy donor (HD) after the application of an αIL-2 neutralizing antibody or the respective isotype control during CD3/CD28 stimulation for 24 h. 1X, 10X, 100X represent the titrated concentration of neutralizing antibodies according to the expected amount of secreted IL-2. MW in kDa (pSTAT5: 90, GAPDH: 37). Colorimetric images depict the used protein ladder (L), while Chemiluminescence images present the proteins of interest. Grey squares highlight the blot area presented in Supplementary Figure 5B. MW: Molecular Weight.

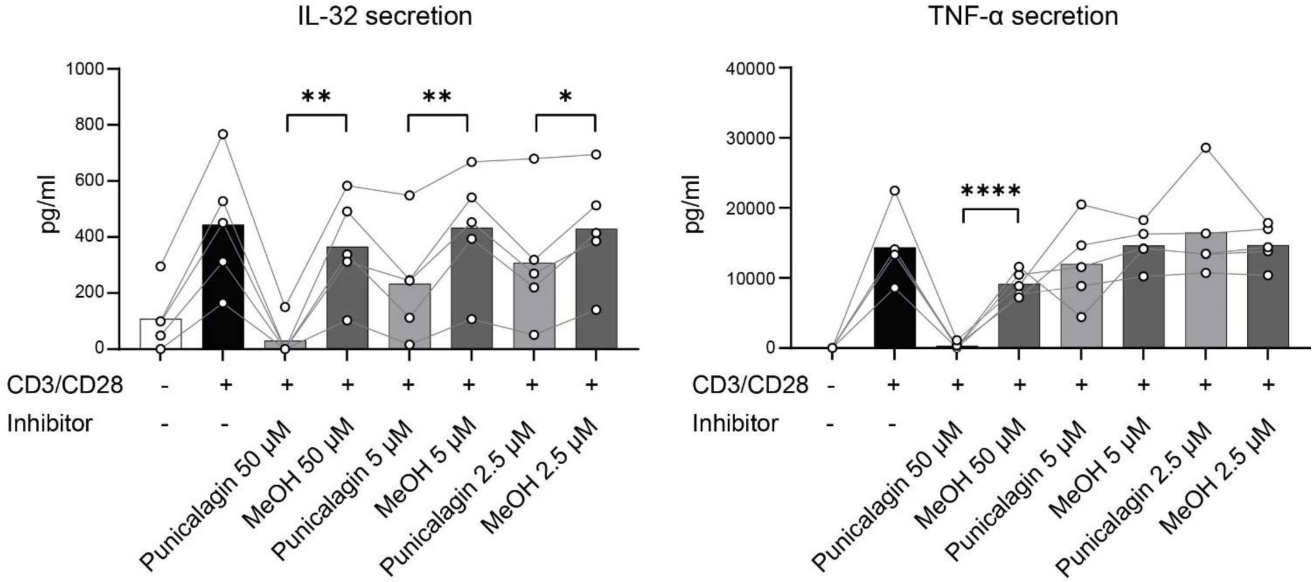

Supplementary Figure 7. Titration of Punicalagin treatment. IL-32 and TNF-α secretion of Survivin T cells after 4 h of CD3/CD28 stimulation in the presence of the secretion inhibitor Punicalagin at 50, 5 or 2.5 µM analyzed by ELISA. Additional treatment with the respective volume of methanol (MeOH), the dissolution medium of Punicalagin, added to the medium for each Punicalagin concentration. Cumulative data of n=5 independent experiments, Student's paired t-test, $^{*}p<0.05$, $^{**}p<0.001$, $^{****}p<0.0001$.

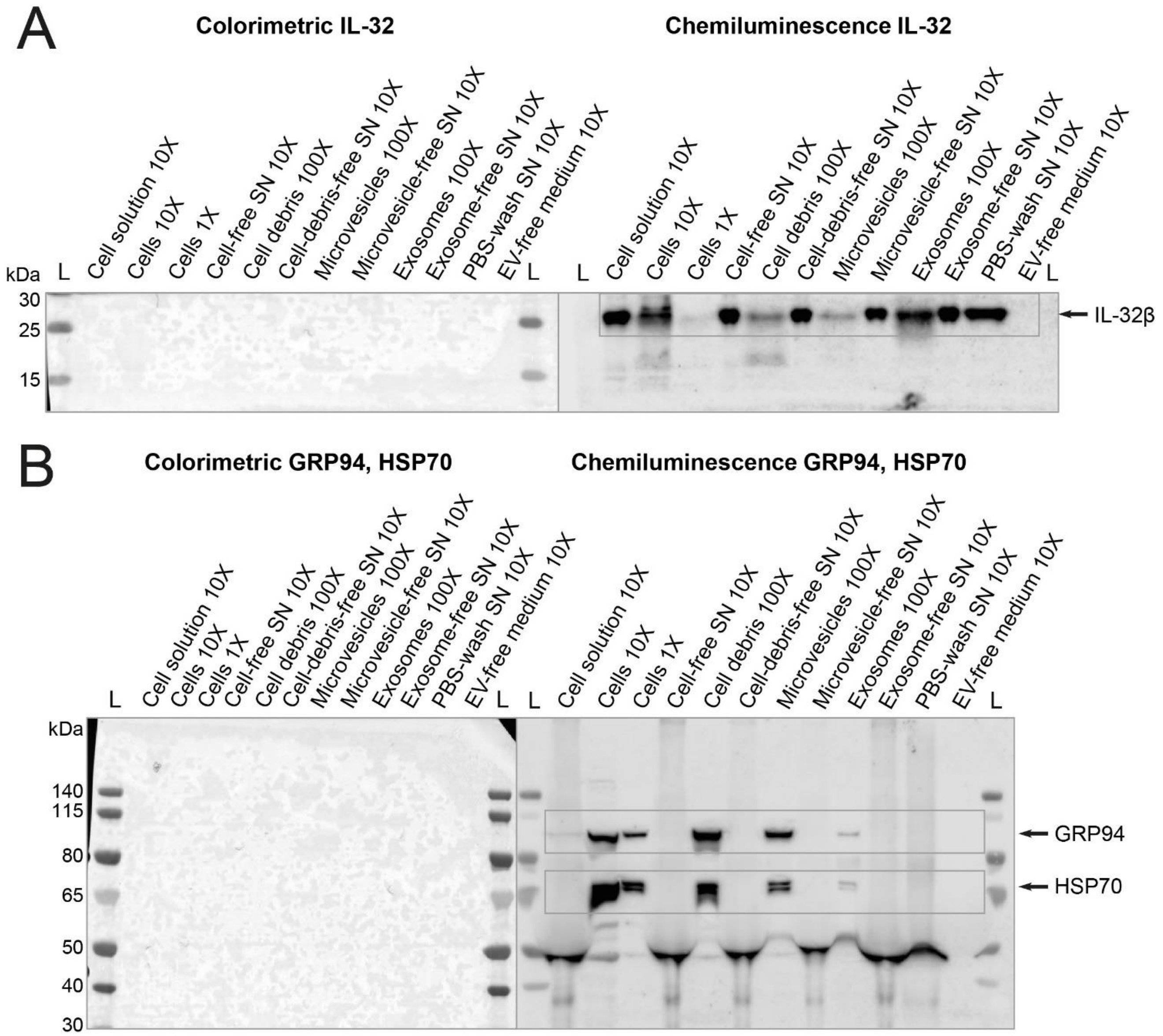

Supplementary Figure 8. Original Western Blot membranes corresponding to main Figure 8A. WB analysis of the expression of **(A)** IL-32β and **(B)** the microvesicle/exosome markers GRP94 and HSP70 in lysates of cells, cell-debris, microvesicles and exosomes, into the respective cell solution and cell-free, cell debris-free, microvesicle-free and exosome-free supernatant (SN), the PBS-wash SN, which was used to wash the exosome pellet, and in the EV-free medium used to culture Survivin-specific T cells for 4 h with anti-CD3/CD28 antibodies. The loaded quantity of each lysate and SN sample corresponds to 0.16×10^6 (1X), 1.6×10^6 (10X) and 16×10^6 (100X) Survivin-specific T cells and the amount of PBS-wash SN derived from this cell number, or the volume of EV-free medium used to culture this cell number. MW in kDa (IL-32β: 23.1, GRP94: 98, HSP70: 70). Colorimetric images depict the used protein ladder (L), while Chemiluminescence images present the proteins of interest. Grey squares highlight the blot area presented in Figure 8A. MW: Molecular Weight.
